# Synergistically Stabilizing Zinc Anodes by Molybdenum Dioxide Coating and Tween 80 Electrolyte Additive for High-Performance Aqueous Zinc-Ion Batteries

**DOI:** 10.1021/acsami.3c08474

**Published:** 2023-11-21

**Authors:** Nhat Anh Thieu, Wei Li, Xiujuan Chen, Qingyuan Li, Qingsong Wang, Murugesan Velayutham, Zane M. Grady, Xuemei Li, Wenyuan Li, Valery V. Khramtsov, David M. Reed, Xiaolin Li, Xingbo Liu

**Affiliations:** † Department of Mechanical and Aerospace Engineering, Benjamin M. Statler College of Engineering and Mineral Resources, West Virginia University, Morgantown, West Virginia 26506, United States; ‡ Bavarian Center for Battery Technology (BayBatt), Department of Chemistry, University of Bayreuth, Universitätsstrasse 30, 95447 Bayreuth, Germany; § In Vivo Multifunctional Magnetic Resonance Center, Robert C. Byrd Health Sciences Center, West Virginia University, Morgantown, West Virginia 26506, United States; ∥ Department of Biochemistry and Molecular Medicine, School of Medicine, West Virginia University, Morgantown, West Virginia 26506, United States; ⊥ Energy and Environmental Directorate, Pacific Northwest National Laboratory, Richland, Washington 99352, United States; # Department of Chemical and Biomedical Engineering, Benjamin M. Statler College of Engineering and Mineral Resources, West Virginia University, Morgantown, West Virginia 26506, United States

**Keywords:** MoO_2_ coating layer, Zn-ion batteries, Tween 80 additive, corrosion inhibitor, Zn anodes

## Abstract

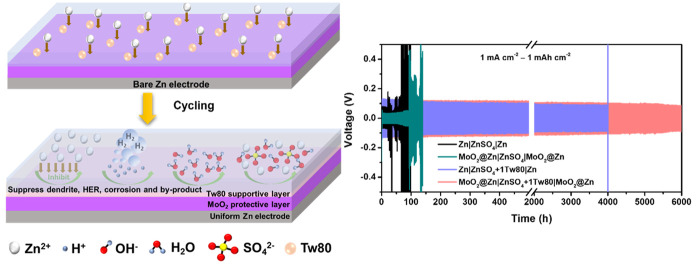

Recently, aqueous zinc-ion batteries (ZIBs) have become increasingly attractive as grid-scale energy storage solutions due to their safety, low cost, and environmental friendliness. However, severe dendrite growth, self-corrosion, hydrogen evolution, and irreversible side reactions occurring at Zn anodes often cause poor cyclability of ZIBs. This work develops a synergistic strategy to stabilize the Zn anode by introducing a molybdenum dioxide coating layer on Zn (MoO_2_@Zn) and Tween 80 as an electrolyte additive. Due to the redox capability and high electrical conductivity of MoO_2_, the coating layer can not only homogenize the surface electric field but also accommodate the Zn^2+^ concentration field in the vicinity of the Zn anode, thereby regulating Zn^2+^ ion distribution and inhibiting side reactions. MoO_2_ coating can also significantly enhance surface hydrophilicity to improve the wetting of electrolyte on the Zn electrode. Meanwhile, Tween 80, a surfactant additive, acts as a corrosion inhibitor, preventing Zn corrosion and regulating Zn^2+^ ion migration. Their combination can synergistically work to reduce the desolvation energy of hydrated Zn ions and stabilize the Zn anodes. Therefore, the symmetric cells of MoO_2_@Zn∥MoO_2_@Zn with optimal 1 mM Tween 80 additive in 1 M ZnSO_4_ achieve exceptional cyclability over 6000 h at 1 mA cm^–2^ and stability (>700 h) even at a high current density (5 mA cm^–2^). When coupling with the VO_2_ cathode, the full cell of MoO_2_@Zn∥VO_2_ shows a higher capacity retention (82.4%) compared to Zn∥VO_2_ (57.3%) after 1000 cycles at 5 A g^–1^. This study suggests a synergistic strategy of combining surface modification and electrolyte engineering to design high-performance ZIBs.

## 1 Introduction

Aqueous metal-ion batteries have many advantages, including higher ionic conductivity, cost-effectiveness, easier processing, and higher safety with aqueous electrolytes, which makes them a promising candidate for energy storage systems.^1^ In this regard, aqueous zinc-ion batteries (ZIBs) have attracted intense interest due to the merits of Zn anodes with a high theoretical capacity (820 mAh g^–1^), low redox potential (−0.76 V vs. standard hydrogen electrode), low cost, inherent safety, low toxicity, and mature recyclability.^2,3^ However, Zn metal anodes undergo dendritic issues due to inhomogeneous Zn^2+^ ion deposition and dissolution on the electrode surfaces.^3^ Moreover, the Zn anode surface and electrolyte interaction can cause electrode corrosion. Corrosion of Zn anodes creates an irregular surface, causing uneven nucleation sites for Zn^2+^ ions and facilitating the hydrogen evolution reaction (HER) on such corroded Zn surfaces.^4^ Then, some passivated byproducts, such as zinc sulfate hydroxide hydrate (Zn_4_SO_4_(OH)_6_·*x*H_2_O), can be formed during the electrochemical process.^3^ Thus, Zn anodes suffer from poor electrochemical stability, low Coulombic efficiency (CE), short circuits, and limited cycle life, restricting the practical development of ZIBs.^5^ Various strategies have been proposed to address these issues of Zn anodes, including modifying the anode surface,^6,7^ optimizing the electrolyte composition,^8,9^ constructing 3D-structured or alloyed anodes,^10,11^ and fabricating functional separators.^12,13^ Among them, the coating and electrolyte engineering strategies offer great potential for the practical application of Zn-ion batteries.

Constructing an artificial interface coating layer on the Zn anode surface effectively inhibits Zn dendrite formation and the electrochemical corrosion of Zn anodes. Two types of surface coating materials are commonly reported, including insulating and conductive materials. The use of nonconductive coating layers on the Zn anode surface, such as metal oxides, polymers, and inorganic compounds, can ensure uniform Zn deposition by guiding Zn^2+^ flux, and prevent corrosion by blocking free water with dissolved oxygen.^6,14,15^ However, the high impedance of interfaces between these nonconductive artificial layers and Zn anodes may restrain the performance of ZIBs at high rates.^7^ On the other hand, many conductive coating materials such as Cu,^7^ Au,^16^ MXene,^17^ and porous carbon network,^18^ can effectively homogenize the surface electric field and Zn^2+^ concentration field, inducing uniform Zn deposition. Although protective layers have effectively improved the electrochemical performance of ZIBs, some unavoidable limitations still restrict their practical applications. For example, surface modifications are usually produced using complex manufacturing processes, which may increase electron/ion transfer resistance. The coating may hinder the transport of ions to the Zn anode surface during Zn deposition. The exfoliation of the coating layer could occur during cycling due to the poor affinity between protective materials and Zn substrates. Furthermore, electrolyte optimization can suppress Zn anode issues, especially by adding various additives to the electrolyte.^8,9,19^ In view of practical applications, the use of an electrolyte additive is a facile method to regulate the Zn anode-electrolyte interface. Adding additives can also stabilize Zn ions in electrolytes, and a homogeneous nucleation process can be achieved by the control of electrode–electrolyte interfaces due to the additives.^20^ Nevertheless, these additives cannot completely hinder Zn anodes from contacting aqueous electrolytes, and some may even consume electrons from Zn anodes and be deteriorated (electrocatalytically oxidized/reduced), thereby decreasing the Coulombic efficiency and reversibility.^19^ To avoid these issues, a protective layer is typically introduced to protect Zn anodes from reacting with electrolyte components.

As a result, an integrated strategy based on the synergistic effect of electrode surface engineering and electrolyte modification is a promising approach for stabilizing the Zn anode. Nevertheless, there is a need for more research on these synergistic effects and mechanisms of modification. This work introduces the integration of a molybdenum oxide coated Zn anode with the addition of Tween 80 additive to ZnSO_4_ electrolyte, giving rise to the synergistic protection for stabilizing the Zn anode. Molybdenum oxide has high chemical stability in acidic electrolytes, multiple metal center valences, and tunable oxygen nonstoichiometry leading to tailored oxygen vacancies,^21,22^ which may be used as an artificial interface coating to engineer the electrochemistry of Zn. Particularly, molybdenum dioxide (MoO_2_) with a metallic nature possesses a high theoretical capacity (838 mAh g^–1^), narrow band gap (≈0.9 eV), outstanding conductivity (1.1 × 10^6^ S·m^–1^ at 300 K in the form of thin film), high chemical and thermal stability, low toxicity, and cost-effectiveness.^23,24^ Due to its one-dimensional (1D) tunnels for fast ion diffusion, MoO_2_ exhibits not only high electronic conductivity but also considerable ionic conductivity.^24^ Although MoO_2_ has a less prominent presence in technology than MoO_3_, it has recently been identified as a promising anode material for lithium ion batteries (LIBs).^24−26^ Since molybdenum (Mo) is a transition metal element with a unique flexibility in valences, nanostructured molybdenum dioxide (MoO_2_) exhibits high Li^+^ storage capacities because of its large redox capability of the multivalent Mo atoms.^25,26^

In this study, MoO_2_ nanoplates were used as the protective coating layer for stabilizing metallic Zn anodes in ZIBs with mildly acidic aqueous electrolytes. Due to the relatively high electrical conductivity of MoO_2_, the coating layer can homogenize the surface electric field and the Zn^2+^ concentration field in the vicinity of the Zn anode, thereby regulating Zn^2+^ ion distribution and inhibiting side reactions. On the other hand, Tween 80 (Tw80), a nonionic surfactant, has been used as a promising corrosion inhibitor for Zn in acidic solution in the corrosion field due to its hydrophobic main chain and abundant hydrophilic groups.^27−29^ Therefore, the adsorption of Tw80 on the Zn surface may alter the corrosion-resistance property and interfacial chemical environment of Zn. Consequently, the MoO_2_ coating layer and Tw80 as an electrolyte additive could synergistically protect the Zn metal anodes for developing stable aqueous ZIBs. This work provides a new method combining the MoO_2_-based coating layer and nonionic surfactant additive for stabilizing Zn anodes for ZIBs.

## 2 Experimental Section

### 2.1 Synthesis of MoO_2_

MoO_2_ was synthesized by thermal reduction from the MoO_3_ nanobelts. The details of the hydrothermal synthesis of MoO_3_ nanobelts are described in the Supporting Information. The as-prepared MoO_3_ was then placed inside a tube furnace (MTI Corporation) at 650 °C for 4 h in a mixture of N_2_ and H_2_ gas flow (95% N_2_ + 5% H_2_). After heating, the system was cooled to room temperature under a gas flow, and the obtained powder was MoO_2_. The other MoO_2_ control samples were prepared by similar thermal reduction for different durations (1, 8, and 12 h).

### 2.2 Preparation of MoO_2_-Coated Zn Anodes

Initially, a coating slurry was prepared by mixing MoO_2_ powder and carboxymethyl cellulose (CMC) with a weight ratio of 8:2 in DI water under magnetic stirring at room temperature overnight to obtain a homogeneous slurry. Next, abrasive papers were used to remove the oxidation layer from the bare Zn foils followed by ultrasonic cleaning with ethanol. Polished Zn foils were further used as either control samples or substrates for MoO_2_ coated Zn. In the following steps, the obtained slurry was cast onto clean Zn foils using a doctor blade method, then vacuum-dried overnight at 60 °C. The obtained MoO_2_-coated Zn foil was denoted as MoO_2_@Zn.

### 2.3 Synthesis of VO_2_ Nanorods and Preparation of Cathodes

A reported hydrothermal method was used to fabricate VO_2_ nanorods.^30^ Typically, 1 g of V_2_O_5_ was added to 30 mL of deionized water and ethylene glycol mixture solution in a 3:2 volume ratio. The suspension mixture was vigorously stirred for 2 h and then was transferred into a Teflon-line autoclave for a hydrothermal reaction at 180 °C for 5 h. The obtained products were centrifuged, washed with deionized water and ethanol several times, and then dried in a vacuum oven at 80 °C overnight.

The cathode was fabricated by mixing VO_2_, super P conductive carbon, and CMC in a weight ratio of 7:2:1 with DI water as a solvent. The slurry was then cast on carbon paper and dried at 80 °C for 24 h in a vacuum oven. After drying, a 12 mm circular disk was punched from the sheet as a VO_2_ cathode.

### 2.4 Electrochemical Measurements

Bare Zn foil and MoO_2_@Zn were cut into 15 mm circular disks as electrodes. Glass microfiber sheets were cut into 16 mm circular disks as separators. The electrolytes used in this work are 1 M ZnSO_4_ and 1 M ZnSO_4_+1 mM Tw80 aqueous solutions, abbreviated as the ZnSO_4_ electrolyte and ZnSO_4_+1Tw80 electrolyte. For comparison, different concentrations (0.5, 5, and 10 mM) of Tw80 in a 1 M ZnSO_4_ aqueous solution were prepared and denoted as ZnSO_4_+0.5Tw80, ZnSO_4_+5Tw80, and ZnSO_4_+10Tw80 electrolytes, respectively. Coin cells (CR2032) were assembled with separators for symmetrical, half, and full cells. The electrochemical performances of these cells were evaluated using multichannel battery testing equipment (LAND CT2001). Symmetrical cells were assembled by using bare Zn or MoO_2_@Zn as working and counter electrodes. Different current densities of 1–10 mA cm^–2^ and an areal capacity of 1–5 mAh cm^–2^ were used for testing symmetric Zn∥Zn and MoO_2_@Zn∥MoO_2_@Zn cells. The Zn∥Cu, MoO_2_@Zn∥Cu, and MoO_2_@Zn∥MoO_2_@Cu half cells were prepared to evaluate the Coulombic efficiencies with Cu as a working electrode (diameter of 15 mm). Full cells were assembled by using bare Zn or MoO_2_@Zn anodes and VO_2_ cathodes. The mass loading of VO_2_ on the cathode is in the range of 1.4–2.1 mg cm^–2^. The full cells were cycled between 0.2 and 1.6 V vs Zn^2+^/Zn. Cyclic voltammetry (CV) curves were conducted on an electrochemical workstation (BioLogic, SP–300). Tafel plots and chronoamperometry (CA) curves were also performed under an electrochemical workstation (BioLogic, SP–300) with a three-electrode system including bare Zn or MoO_2_@Zn (1 cm × 1 cm) as working electrode, Pt wire as counter electrode, and saturated calomel electrode (SCE) as a reference electrode. The Tafel plots were recorded with a potential range of −0.3 to 0.3 V vs open circuit voltage (OCV) of the system at a scan rate of 1 mV s^–1^. An overpotential of −150 mV vs Zn^2+^/Zn was applied for testing CA curves. An optical microscope system (Keyence VHX–7000) recorded the homemade transparent cells’ plating/stripping process under an electrochemical workstation (Gamry Interface 5000E). To determine the activation energy (*E*_a_) of the Zn deposition process, electrochemical impedance spectroscopy (EIS) tests of symmetric cells at different temperatures were carried out with the amplitude of 5 mV in the frequency range from 1 MHz to 0.05 Hz using a potentialstat (BioLogic VSP). The charge transfer resistance (*R*_ct_) was obtained by fitting the EIS curves with the modified Randles equivalent circuit model by using EC-Lab software. Then, eq 1 was used to calculate the *E*_a_ values, according to the Arrhenius equation:
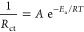
1where *R*_ct_ is the charge transfer resistance obtained from the EIS spectra, *R* is the gas constant, and *T* is the thermodynamic temperature.

## 3 Results and Discussion

**Figure 1:**
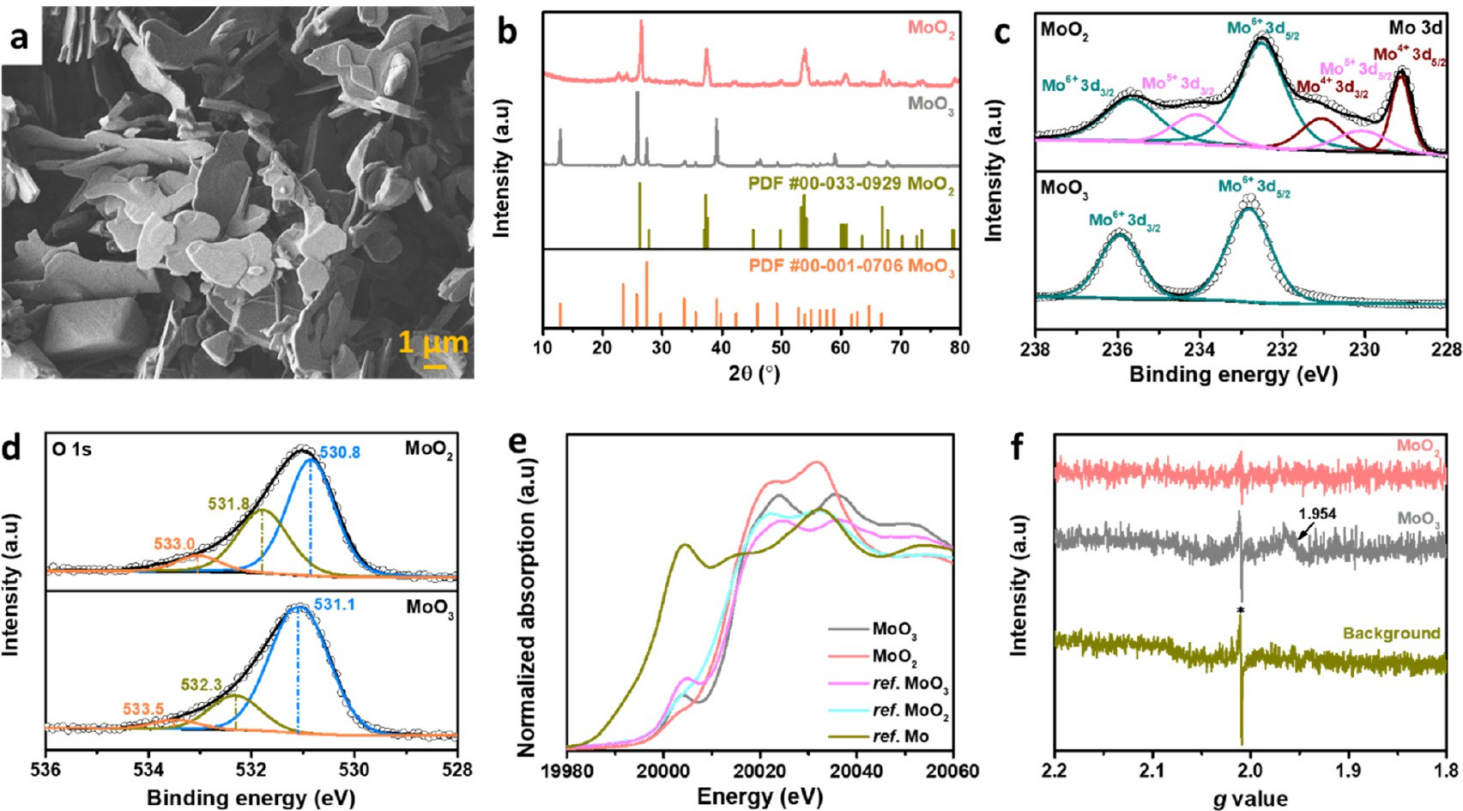
(a) SEM image of synthesized MoO_2_. (b) Corresponding XRD patterns of MoO_3_ and MoO_2_. XPS spectra of MoO_3_ and MoO_2_ in (c) Mo 3d and (d) O 1s spectrum. (e) Mo K-edge XANES spectra of the synthesized MoO_3_ and MoO_2_ with the reference spectra of MoO_3_, MoO_2_, and Mo. (f) EPR spectra of MoO_3_ and MoO_2_ and background signals from the Dewar.

The MoO_2_ was prepared by the thermal reduction of the hydrothermally synthesized MoO_3_ precursor in a H_2_/N_2_ atmosphere. The morphology changes from MoO_3_ to MoO_2_ can be observed by a scanning electron microscope (SEM), as shown in Figures 1a and S1. After the thermal treatment, MoO_3_ nanobelts are converted to MoO_2_ nanoplates, favorable for the rapid diffusion of metal ions.^31^ The X-ray diffraction (XRD) pattern shows that MoO_3_ was reduced to MoO_2_ at 650 °C (Figure 1b). There are high index diffraction peaks observed at 2θ ≈ 26.5°, 37.5°, and 54.0° associated with reflection planes (1̅11), (2̅11), and (2̅22) of MoO_2_, which are similar to the previous report.^32^ The sharp peaks show that the obtained monoclinic MoO_2_ from the reduction process possesses a high crystallinity. Further, X-ray photoelectron spectroscopy (XPS) analysis is used to verify the reduction of MoO_3_ to MoO_2_. The XPS survey spectra reveal the presence of Mo and O elements in both the MoO_3_ and MoO_2_ samples (Figure S2). A pair of peaks corresponding to Mo^6+^ are found in the XPS spectrum of Mo 3d in MoO_3_ (Figure 1c). Also, Figure 1c illustrates that the Mo 3d region of MoO_2_ contains three components, Mo^6+^, Mo^5+^, and Mo^4+^, consistent with the previous report.^33^ The multivalent Mo in MoO_2_ indicates the reduction of MoO_3_ to MoO_2_. On the other hand, the O 1s spectrum of MoO_3_ exhibits the prominent peak at 531.1 eV, which corresponds to lattice oxygen (O^2–^), and the other two peaks (532.3 and 533.5 eV) are attributed to surface absorbed species (OH^–^, O^–^) (Figure 1d).^34^ It is observed that the characteristic peaks of Mo^6+^ and the entire O 1s peak of MoO_2_ shifts to a lower level compared to that of MoO_3_, indicating that the coordination environment of Mo with O has changed (Figure 1c,d).^34^ XPS results indicate that MoO_2_ may contain a mixture of Mo oxidation states between +6 and +4. This electronic configuration achieves a large electrical conductivity due to the concentration of free carriers, which effectively improves the electrochemical properties.^25^

X-ray absorption spectroscopy (XAS) measurement is then performed in the Mo K-edge region for the as-prepared MoO_3_ and MoO_2_ samples to confirm the reduction of MoO_3_ to MoO_2_. A comparison of the X-ray absorption near edge structure (XANES) spectra of MoO_3_ and MoO_2_ is shown in Figure 1e. Accordingly, the XANES spectrum of the as-prepared MoO_3_ shows the pre-edge absorption at ≈20004.2 eV, almost identical to the reference MoO_3_ (≈20004.9 eV), suggesting a similar structure to orthorhombic α-MoO_3_.^35^ The pre-edge is associated with the electronic transition from 1s to 4d in systems with tetragonal symmetry, such as MoO_3_, and is not observed in MoO_2_ because Mo is coordinated by regular octahedra.^36^ The pre-edge peak of the synthesized-MoO_2_ is significantly reduced, and the absorption edge shifts to lower energy, resulting in a spectrum closely resembling monoclinic MoO_2_.^36^ The reason is that a phase transition occurs from orthorhombic α-MoO_3_ with a distorted octahedral geometry to symmetric H_*y*_MoO_3–*x*_ suboxide with low-valence Mo atoms.^36^ These results indicate the reduction of Mo^6+^ ions in MoO_3_ to lower oxidation states (Mo^5+^, Mo^4+^) in MoO_2_ due to the removal of oxygen and accompanied by partial reduction. This spectral change is consistent with both XRD and Mo 3d XPS results. Additionally, MoO_3_ shows a broad electron paramagnetic resonance (EPR) peak at *g* = 1.954 (Figure 1f), which corresponds to EPR active Mo^5+^ centers/species. The hyperfine coupling/splitting corresponds to Mo isotopes with I = 5/2 (natural abundance) is not resolved.^37^ However, no EPR signal was observed for the MoO_2_ sample. This result suggests that this sample contains more than one ferromagnetic cluster/domain, which is due to the ferroelectric/metallic materials of MoO_2_ in nature.^38^ The magnetic interaction between the centers leads to ferromagnetic coupling and the metallic nature of the materials. The metallic nature of the materials did not allow the microwave to pass through the samples in the EPR cavity and dampened the resonance condition. Therefore, the MoO_2_ sample showed no EPR signal.

**Figure 2:**
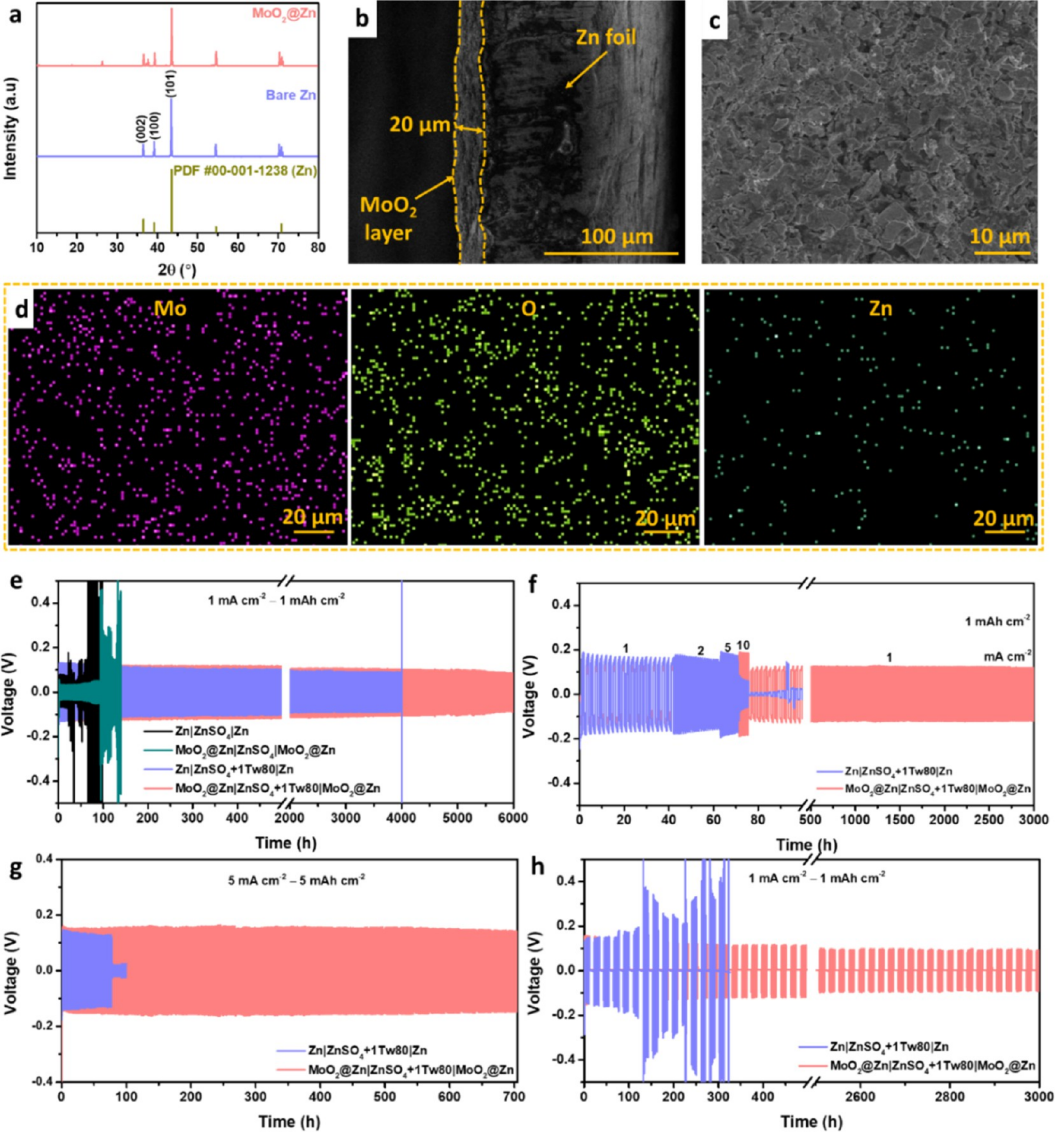
(a) XRD patterns of bare Zn and MoO_2_@Zn. SEM images of MoO_2_@Zn at the (b) cross-section and (c) surface with (d) corresponding EDS mapping. (e) Cycling performance of bare Zn and MoO_2_@Zn symmetric cells using ZnSO_4_ and ZnSO_4_+1Tw80 electrolytes at 1 mA cm^–2^–1 mAh cm^–2^. (f) Rate performance of symmetric cells using ZnSO_4_+1Tw80 electrolyte. (g) Cycling performance of bare Zn and MoO_2_@Zn symmetric cells using ZnSO_4_+1Tw80 electrolyte at a high current density–capacity of 5 mA cm^–2^–5 mAh cm^–2^. (h) Voltage profiles of bare Zn and MoO_2_@Zn symmetric cells with ZnSO_4_ electrolyte containing Tw80 additive for testing under alternate 10 continuous stripping/plating cycles and 10 h resting.

A MoO_2_ layer was then coated on polished Zn foil (MoO_2_@Zn) by a facile doctor-blade method. Figure 2a shows MoO_2_@Zn maintaining identical crystallographic orientation as bare Zn foil with characteristic peaks of MoO_2_ at 2θ ≈ 26.5° and 37.6°. While the original bare Zn has several ridges and holds on the surface (Figure S3), MoO_2_@Zn demonstrates a surface densely covered with MoO_2_ nanoplates with a thickness of 20 μm shown in the SEM images and energy dispersive X-ray spectroscopy (EDS) mapping (Figure 2b–d). The electrochemical stability of Zn in 1 M ZnSO_4_ is then investigated, focusing on the effects of the MoO_2_ coating and Tw80 electrolyte additive, as 1 M ZnSO_4_ is more industrially relevant than costly zinc triflate and other high-concentration electrolytes. The symmetric cells were assembled to evaluate the cycling stability of Zn plating/stripping at different current densities and capacities. Figure 2e exhibits the long-term cycling of bare Zn and MoO_2_@Zn symmetric cells with and without Tw80 electrolyte additives at 1 mA cm^–2^ for 1 mAh cm^–2^. In a blank ZnSO_4_ electrolyte, the Zn symmetric cell maintains a short lifespan for less than 50 h, while the MoO_2_@Zn symmetric cell exhibits improved stability for ca. 100 h. Despite the longer lifetime of symmetric cells provided by MoO_2_@Zn electrodes compared with bare Zn electrodes, the cycle life has not been significantly improved. With the addition of 1 mM Tw80 in the ZnSO_4_ electrolyte, the Zn symmetric cell shows excellent stability up to 4000 h. Nevertheless, the bare Zn electrodes remain directly in contact with water in the electrolyte, resulting in a possibility of HER forming or corrosion, which would adversely affect cell performance. Interestingly, the MoO_2_@Zn symmetric cell with the Tw80 electrolyte additive achieves an extended cycle life of 6000 h. It is observed that bare Zn and MoO_2_@Zn electrodes show an obvious Zn dendrite after 50 cycles in the blank ZnSO_4_ electrolyte (Figure S4a,d), while no apparent dendrites are observed on the surface of bare Zn and MoO_2_@Zn in ZnSO_4_+1Tw80 electrolyte after 50 cycles (Figure S4b,e). As exhibited in the XRD pattern (Figure S4c), a prominent peak at 2θ ≈ 8.07° and other peaks at 2θ ≈ 16.22° and 24.44° of Zn_4_SO_4_(OH)_6_·*x*H_2_O (ZSOH) are observed for the bare Zn electrode after 50 cycles in blank ZnSO_4_ electrolyte, while applying the MoO_2_ coating layer could reduce the ZSOH intensity peaks (Figure S4f). On the other hand, no obvious ZSOH could be found on the XRD patterns of both electrodes in the ZnSO_4_+1Tw80 electrolyte after 50 cycles. Based on the results, MoO_2_ alone can slightly prevent the formation of byproducts, whereas combining MoO_2_@Zn with the Tw80 additive can significantly inhibit corrosion. Therefore, the combination of using the MoO_2_ coating layer incorporating the Tw80 additive can ensure the stability of Zn electrodes.

Figure S5 compares the voltage profile between bare Zn and MoO_2_@Zn symmetric cells in the ZnSO_4_+1Tw80 electrolyte. In the initial cycle, the voltage hysteresis of two cells displays the same value (∼110 mV). As the cycle number increased, the voltage of the MoO_2_@Zn symmetric cell remains relatively stable, while the voltage of the bare Zn cell slightly drops after 100 cycles. At cycle 2001, a slight drop to 95.8 mV could be observed in the MoO_2_@Zn cell, and the bare Zn cell shows a polarization. Moreover, the rate performance of bare Zn and MoO_2_@Zn symmetric cells using ZnSO_4_+1Tw80 electrolyte is evaluated at different current densities from 1 to 10 mA cm^–2^ with a fixed capacity of 1 mAh cm^–2^ (Figure 2f). A sudden short circuit occurs in the Zn symmetric cell when the current density is increased to 10 mA cm^–2^. In contrast, the MoO_2_@Zn symmetric cell exhibits excellent performance even at 10 mA cm^–2^. Afterward, when the current density is returned to 1 mA cm^–2^, the MoO_2_@Zn symmetric cell shows an excellent reversible stability of 3000 h with prominent resilience. The MoO_2_@Zn symmetric cells also demonstrate higher stability than Zn symmetric cells when the current density increases to 5 mA cm^–2^ (Figure 2g) and 10 mA cm^–2^ (Figure S6) at a fixed capacity of 5 mAh cm^–2^. These results indicate the great protective effect of the Tw80 additive and MoO_2_ coating on Zn electrodes. Furthermore, the shelving and restoring abilities of symmetric cells with bare Zn and MoO_2_@Zn anodes containing ZnSO_4_+1Tw80 electrolyte are also compared by alternate cycling and resting evaluation (Figure 2h). The symmetric cell of MoO_2_@Zn is stable for 3000 h with an average overpotential of 110 mV. Conversely, the bare Zn anode produces an apparent fluctuation in the polarization voltage, which culminates in cell failure after 320 h. When switching between cycling and resting conditions, the MoO_2_@Zn electrode shows a stable voltage response, indicating excellent corrosion resistance and resilience. Thus, the MoO_2_@Zn anode using the ZnSO_4_+1Tw80 electrolyte additive extensively stabilizes the Zn electrode and ensures a prolonged life for the cell. Table S1 compared our work’s test conditions and electrochemical performance with those previously reported by others, indicating that the MoO_2_@Zn electrode with Tw80-assisted electrolyte delivers superior and competitive performance at a wide range of current density-capacity in symmetric cells.

To verify the effect of Tw80 on regulating the electrode–electrolyte interface, experiments using MoO_2_@Zn symmetric cells were conducted. With a fixed concentration of 1 mM Tw80, the concentration of ZnSO_4_ is increased to 2 M to explore the effect of the salt concentration on the system. According to Figure S7a, the lifespan of the MoO_2_@Zn symmetric cell using 2 M ZnSO_4_ shows a stability performance of over 1000 h, six times less than that of the cell using 1 M ZnSO_4_. It is known that increasing the electrolyte concentration could result in a decrease in solvent molecules surrounding the Zn^2+^ ions, altering the solvation structures and the transport of cations and anions.^39,40^ Increased concentrations also increase the viscosity of the solution, leading to a decrease in the ionic conductivity in the electrolyte. These reasons could account for the shorter performance of symmetric cells using 2 M ZnSO_4_ than that using 1 M ZnSO_4_. A further investigation on Tw80 concentration is explored to verify the optimization of Tw80 concentration varied from 0.5 to 10 mM (0.5Tw80 to 10Tw80) using MoO_2_@Zn symmetric cells at 5 mA cm^–2^–5 mAh cm^–2^. As shown in Figure S7b, the MoO_2_@Zn symmetric cell using ZnSO_4_+1Tw80 exhibits exceptional stability for 705 h. With a slight decrease in Tw80 content to 0.5 mM, the cell also performs well at 650 h. However, the lifespan of cells significantly decreases as the concentration of Tw80 increases to 5 and 10 mM. Figure S8 demonstrates the XRD patterns at the plating (P) and stripping (S) states of MoO_2_@Zn symmetric cells using different Tw80 concentrations after 100 cycles. At the plating state, the cell using 1 mM Tw80 shows the highest intensity of the MoO_2_ characteristic peak at 2θ ≈ 26.2°, meaning that the MoO_2_ coating on the Zn surface maintains a more incredible amount compared to the other cells. In addition, the formation of ZSOH byproduct is discovered at the stripping state. Although ZSOH is discovered in all cells, the cell using 1 mM Tw80 demonstrates the smallest amount of ZSOH. The reason is that a lower concentration (0.5 mM) may not sufficiently suppress water-induced corrosion because of the uncontrollable nucleation process caused by the partially covered Zn foil and incompletely complexed Zn^2+^ ions.^39^ Nevertheless, excessive Tw80 additives (5 and 10 mM) could result in increasing voltage hysteresis. A high energy barrier may exist for Zn deposition due to the smaller ionic conductivity and an adsorption layer caused by the excess Tw80 content.^19^ Thus, the content of additives in the electrolyte should be as small as possible to prevent deterioration of the energy density. Figure S9 shows the corresponding morphology of the MoO_2_@Zn electrode by using different Tw80 concentrations after cycling. After 100 cycles, the MoO_2_@Zn electrode using 1 mM Tw80 maintains a dense MoO_2_ coating layer on the surface. However, for other electrodes using 0.5, 5, and 10 mM Tw80, some Zn or ZSOH flakes appear on the top surface. Consequently, 1 M ZnSO_4_ incorporating 1 mM Tw80 is optimal for the stability of Zn electrodes.

MoO_2_ was selected due to its excellent chemical stability in mildly acidic electrolytes and relatively high electronic conductivity. To better understand the unique effects of combining the MoO_2_-coating layer and Tw80 electrolyte additive on the electrochemical performance improvement, TiO_2_, and WO_2_ were chosen as the alternative coating materials on Zn anodes in the presence of the Tw80 additive. The stability of symmetric cells of TiO_2_ and WO_2_ coated Zn was conducted at high current densities of 5 mA cm^–2^ and 10 mA cm^–2^ with a fixed areal capacity of 5 mAh cm^–2^ using the ZnSO_4_+1Tw80 electrolyte, as shown in Figure S10. The cycling stability of TiO_2_@Zn and WO_2_@Zn symmetric cells at both current densities is inferior to that of MoO_2_@Zn symmetric cells. Furthermore, TiO_2_@Zn cells seem to show a higher polarization. Although both have good chemical stability in acidic solutions, TiO_2_ is a semiconductor with relatively low electrical conductivity at room temperature, while WO_2_ is an analogue of MoO_2_ with relatively high electrical conductivity among metal oxides.^41,42^ The low electrical conductivity of TiO_2_ may not effectively help to make the electric field distribution uniform over the coated Zn surface and thus leads to lower stability and higher polarization at high current densities. The chemistries of MoO_2_ and WO_2_ have many similarities, however, WO_2_ is less stable in acidic solutions than MoO_2_.^43^ Therefore, the relatively high electrical conductivity of WO_2_ can reduce the coated cell polarization but cannot ensure high cycling stability. Therefore, in this work, MoO_2_ has the unique features of high chemical stability and relatively high electrical conductivity, which makes MoO_2_ a promising coating material when combined with the use of the Tw80 additive. Also, MoO_2_ is known for its good electrical conductivity, which makes it potentially useful in battery applications.^23,24,44^ The effect of the electrical conductivity of MoO_2_ on Zn anode protection was evaluated by comparing the electrical conductivities of MoO_2_ and MoO_3_ and the electrochemical stability of Zn anodes coated with them. The MoO_2_ pellet shows a conductivity range of 23–24 S cm^–1^ at room temperature, while the MoO_3_ pellet exhibits a conductivity of less than 3.26 × 10^–13^ S cm^–1^. This result is consistent with the finding that MoO_2_ has a metallic conducting property with a high electrical conductivity, while MoO_3_ has a semiconductor property with a low electrical conductivity.^45^ Similar to other conductive coating materials for Zn anodes, the conductive MoO_2_ coating may homogeneously regulate Zn^2+^ flux and maintain a stable electric field on Zn anode surface to optimize Zn plating/stripping processes.^7,16,17^ The electrochemical stabilities of MoO_3_ and MoO_2_-coated Zn symmetric cells are compared. As shown in Figure S11, the MoO_3_@Zn symmetric cell shows higher polarization and a much shorter cycling lifetime than the MoO_2_@Zn counterpart.

The extent of reduction of the MoO_2_ coating layer also plays a crucial role in the electrochemical stability of Zn. Symmetric cells MoO_2_@Zn with different reduction times (1, 4, 8, and 12 h) were assembled with ZnSO_4_+1Tw80 electrolyte to investigate their electrochemical performances. The symmetric cell using MoO_2_(4 h)@Zn delivers the highest stability at 1 mA cm^–2^ and 5 mA cm^–2^ (Figure S12). Figure S13a illustrates that reduced MoO_2_ at 1 h exhibits the mixture of MoO_3_ and MoO_2_ phases. With the increase in reduction time, the MoO_2_ phase becomes dominant, and the characteristic peaks of MoO_3_ are reduced (at 4, 8, and 12 h). Meanwhile, EPR results show that MoO_2_ samples at longer reduction times also contain more than one ferromagnetic cluster/domain similar to MoO_2_(4 h), demonstrating that different thermal reduction treatment time could change the oxygen coordination of Mo^5+^ center/site in MoO_2_ (Figure S13b). Also, the morphology of MoO_2_ becomes more coarsened with increasing reduction time (Figures 1a and S13c–e). The increased agglomeration results in a decreased surface area of the reduced MoO_2_, leading to the reduction of electrochemical performance.^46^ As a result, symmetric cells of MoO_2_(4 h)@Zn exhibit the most prolonged cycling stability at both low and high current densities, suggesting excellent reversibility of Zn plating/stripping. It is proposed that MoO_2_(4 h) is optimal for both high capacity and stable cyclability, attributed to the smallest crystallite size or the lowest crystalline nature. Therefore, despite the dominant phase of MoO_2_(8 h) and MoO_2_ (12 h) being MoO_2_, the symmetric cells exhibit a higher overpotential and shorter stability than MoO_2_(4 h), implying that the tailored surface structure could affect the energy barrier for the Zn^2+^ ions against desolvation and deposition.^47^ This will be discussed later.

**Figure 3:**
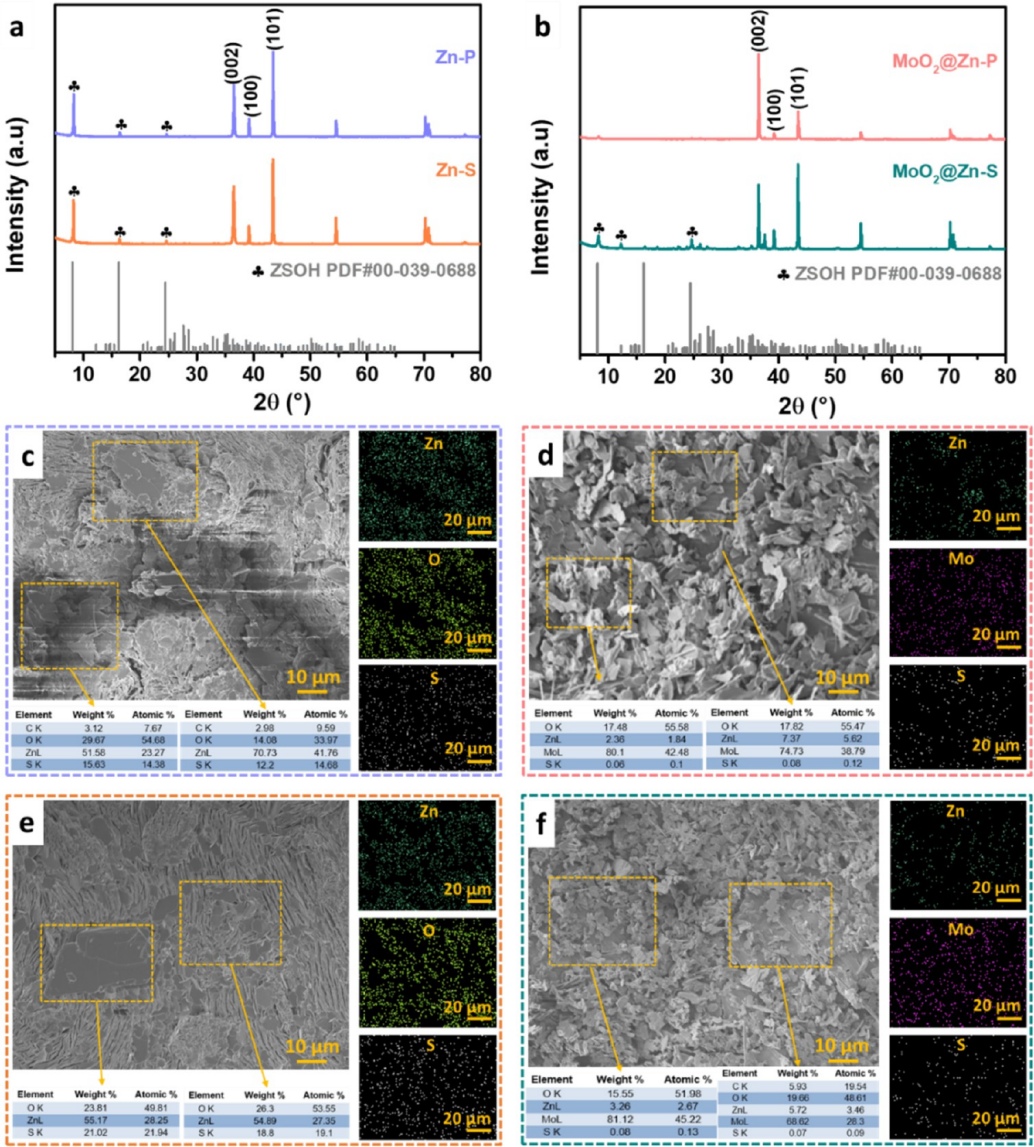
XRD patterns of cycled (a) bare Zn and (b) MoO_2_@Zn electrodes at plating (P) and stripping (S) states. SEM images and EDS analysis after cycling for 500 cycles at the plating state of (c) bare Zn and (d) MoO_2_@Zn. SEM images and EDS analysis after cycling for 500 cycles at stripping state of (e) bare Zn and (f) MoO_2_@Zn. The cells were cycled in a ZnSO_4_+1Tw80 electrolyte.

To explore the interfacial reactions that result in the formation of byproduct and dendrite, the phases of cycled electrodes at plating and stripping states after longer cycling were examined using XRD. As exhibited in the XRD pattern (Figure 3a), a prominent peak at 2θ ≈ 8.07° and other peaks at 2θ ≈ 16.22° and 24.44° of ZSOH are also discovered at plated and stripped forms of bare Zn electrode after 500 cycles. Conversely, no obvious peaks of ZSOH are observed at the plating state of MoO_2_@Zn, while weak ZSOH peaks at 2θ ≈ 8.07°, 12.23°, and 24.44° are detected at the stripping state (Figure 3b). XRD analysis reveals significant differences between cycled Zn electrodes and MoO_2_@Zn electrodes in the crystallographic orientation after plating. While the plated MoO_2_@Zn electrode shows the dominance of the (002) plane to form Zn deposits with horizontal orientation preferentially, the plated bare Zn electrode exhibits the (101) crystal facet exposure and significantly increased the (100) plane, which is easy to form dendrite deposition.^48^ It is also observed that the dominance of (101) orientation for the pristine MoO_2_@Zn sample (Figure 2a) is preserved after the complete stripping of the MoO_2_@Zn electrode, which suggests that the Zn plating/stripping process is highly reversible.^15^

SEM morphology analysis of cycled electrodes also confirms the hypothesis based on the XRD results. As shown in Figure S3, the inherent surface defects of bare Zn, such as holes, are detrimental to the Zn plating/stripping stability since they can capture hydrogen gas generated from corrosion, thereby promoting the HER, and inducing the inhomogeneous local electric field due to the high surface energy and inhomogeneity. Consequently, several flakes appear on the surface of the bare Zn anode after 500 cycles, indicating uneven Zn plating and stripping (Figure 3c,e). In contrast, these protrusions are not evident on the surface of the MoO_2_@Zn electrodes (Figure 3d,f). The EDS elemental maps display that Mo and the O elements dominate the MoO_2_@Zn after cycling, while the surface of bare Zn anode after cycling contains S elements (14:21 at. %) arising from ZSOH. Thus, the MoO_2_ coating layer serves as a stable protective layer on Zn surface to minimize the direct contact between Zn electrodes and the electrolyte and suppress side reactions.

**Figure 4:**
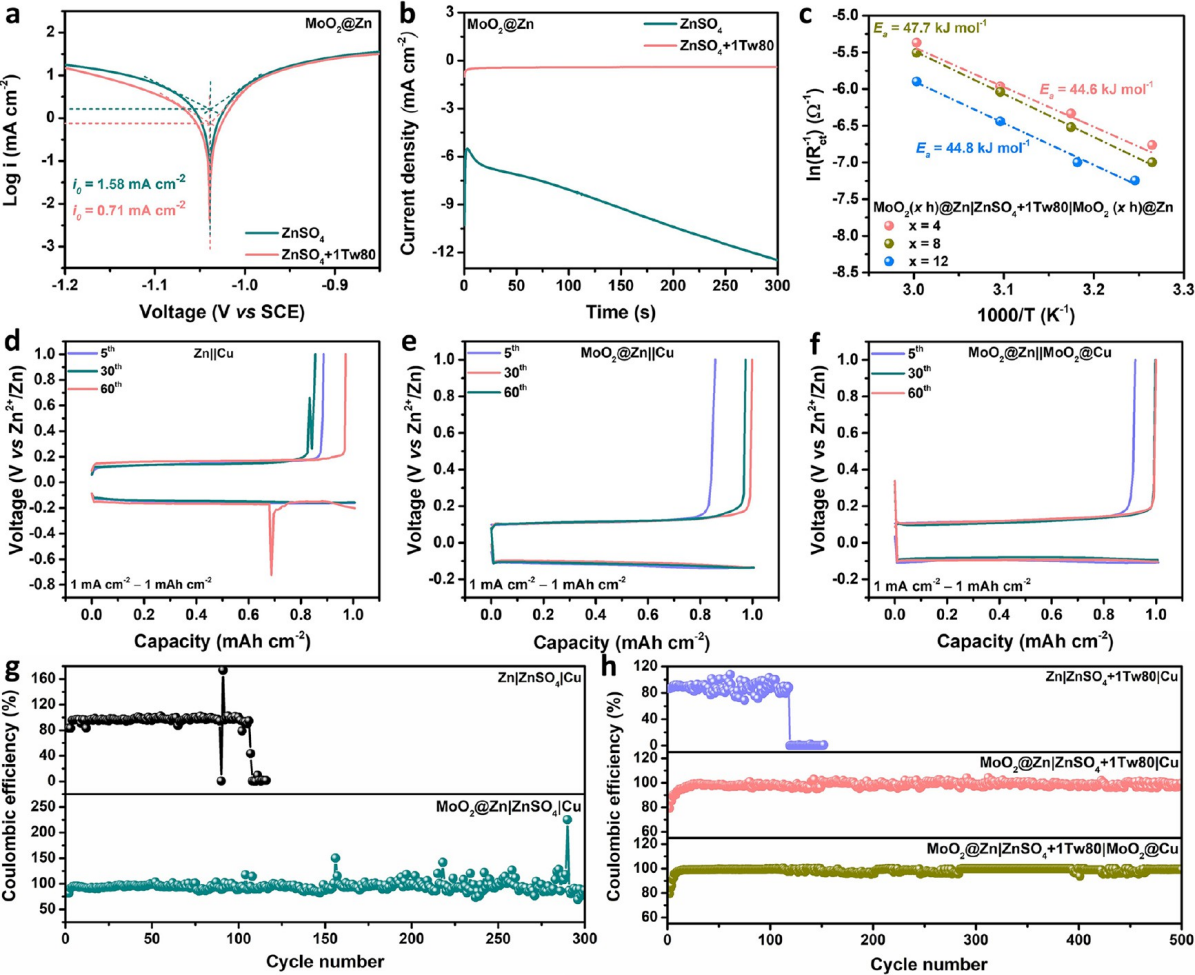
(a) Tafel plots of MoO_2_@Zn electrodes in blank ZnSO_4_ and ZnSO_4_+1Tw80 electrolytes. (b) CA curves of MoO_2_@Zn electrodes in blank ZnSO_4_ and ZnSO_4_+1Tw80 electrolytes. (c) Calculated activation energy of Zn^2+^ desolvation of different MoO_2_ coating layers via the Arrhenius equation. Voltage-capacity profiles of (d) Zn||Cu, (e) MoO_2_@Zn||Cu, and (f) MoO_2_@Zn||MoO_2_@Cu half cells. Coulombic efficiency of (g) Zn||Cu, MoO_2_@Zn||Cu in blank ZnSO_4_ electrolyte and (h) Zn||Cu, MoO_2_@Zn||Cu, and MoO_2_@Zn||MoO_2_@Cu half cells in ZnSO_4_+1Tw80 electrolyte.

The exchange current density (*i*_0_) values of bare Zn and MoO_2_@Zn in different electrolytes were explored via the Tafel plots to determine the regulation of Zn^2+^ ion kinetics.^49^ Note that a low *i*_0_ suggests the suppressed corrosion and Zn plating-stripping kinetics simultaneously, as both involve the similar electrochemical Zn redox reactions.^50^ Therefore, a proper *i*_0_ is preferred to achieve the trade-off. As shown in Figures 4a and S14, the addition of Tw80 can lower *i*_0_ values for bare Zn and MoO_2_@Zn, suggesting that Tw80, as a corrosion inhibitor, can restrain the corrosion of Zn. On the other hand, the MoO_2_ coating layer can increase *i*_0_ in both electrolytes (ZnSO_4_ and ZnSO_4_+1Tw80), suggesting that MoO_2_ can improve the electrochemical redox kinetics of electrolyte–anode interface which is favorable for plating and stripping of Zn anodes.^51^ Therefore, the combination of Tw80 and MoO_2_ coating can help to achieve a balance between inhibiting corrosion and ensuring sufficient electrochemical redox kinetics of the electrolyte–anode interface. To investigate the stability of electrodes in the electrolytes under resting conditions, bare Zn and MoO_2_@Zn plates were immersed in aqueous ZnSO_4_-based electrolytes with or without Tw80 to reveal the corrosion inhibition capability of the coating layer combined with Tw80 (Figure S15). Upon soaking in electrolyte for 30 days, the MoO_2_@Zn surface in blank ZnSO_4_ is severely corroded as evidenced by the color change from brown to white, while the surface of MoO_2_@Zn in ZnSO_4_+1Tw80 is negligibly changed (Figure S16).

CA curves are further analyzed to determine changes in reaction kinetics when Zn^2+^ is deposited under a constant overpotential of −150 mV. In a blank ZnSO_4_ electrolyte, the CA curve of MoO_2_@Zn exhibits an initial decrease in the cathodic current density in the first seconds and then shows the increase of the current density with the cathodic Zn deposition time (Figure 4b). This initial decrease (minus sign only represents the cathodic current direction) is caused due to the formation of a depletion layer close to the working electrode.^52,53^ Then, the continuous increase of the current density is due to the increased surface area caused by the growth of deposited Zn. The results imply that only the MoO_2_-coating layer could not fully hinder the dendritic formation of electrodeposited Zn. On the other hand, a smaller initial current density is observed for bare Zn, and the current density increases over time in blank ZnSO_4_ electrolyte (Figure S17a), indicating that Zn dendrites are forming and gradually growing on the surface of the Zn anode.^54,55^ Moreover, bare Zn in ZnSO_4_+1Tw80 electrolyte (Figure S17b) shows an initial decrease and later increase in current density similar to MoO_2_@Zn in blank ZnSO_4_ electrolyte, indicating that only the Tw80 additive could not fully impede Zn dendrites. However, the current density of MoO_2_@Zn in ZnSO_4_-containing Tw80 electrolyte decreases in a few seconds and then demonstrates a steady current–time behavior (Figures 4b and S17b),^54,56,57^ confirming the promotion of uniform Zn deposition and effective inhibition of Zn dendrite growth in the plating process of Zn^2+^ ions by combining the MoO_2_ coating layer and Tw80 electrolyte additive.

Additionally, the influences of the MoO_2_ coating layer and Tw80 additive on the Zn anode interfaces were evaluated. Contact angle measurements were conducted to examine the effects of the MoO_2_ coating and Tw80 additive on the wettability of Zn. Figure S18a illustrates that the bare Zn exhibited a relatively large contact angle of 87.8° in a ZnSO_4_ solution. The addition of Tw80 in ZnSO_4_ solution reduced the contact angle to 47.9° (Figure S18b), enhancing the hydrophilicity of the Zn surface. Thus, Tw80, as a nonionic surfactant, can help to improve the surface wettability between Zn electrode and electrolyte,^58^ which may assist in the uniform distribution of Zn^2+^ ions. Meanwhile, the MoO_2_ coating layer plays a more pronounced role in significantly reducing the contact angle to almost 0° in the solutions with and without Tw80 (Figure S18c,d), making the coated Zn surface superhydrophilic in the electrolyte possibly by enhancing the surface roughness or heterogeneity.^59,60^ It has been reported that the electrode wettability plays a role in the final Zn deposition pattern and high surface hydrophilicity will result in more uniform Zn^2+^ flux across the surface of MoO_2_@Zn anodes, contributing to homogeneous Zn nucleation and growth.^61^ Consequently, the MoO_2_ coating provides improved electrolyte–anode interfaces and hence enhances Zn^2+^ transport kinetics.

Furthermore, MoO_2_ coating can significantly enhance surface hydrophilicity to improve the wetting of electrolyte on the Zn electrode, which is beneficial for reducing the desolvation energy of hydrated Zn ions.^61^ To determine the activation energy of Zn^2+^ desolvation under the protection of diverse MoO_2_ coatings, EIS measurements of diverse MoO_2_@Zn symmetric cells in ZnSO_4_+1Tw80 electrolyte were conducted at the temperature range from 30 to 60 °C. At a given temperature, the EIS curves show that the charge-transfer resistance (*R*_ct_) increased with the extended reduction time of MoO_2_ (Figure S19a–c), suggesting improved charge transfer kinetics in the MoO_2_(4 h)@Zn cells. Based on the Arrhenius equation (Supporting Information), the desolvation activation energy (*E*_a_) can be obtained. The calculated activation energy (*E*_a_) of Zn^2+^ desolvation with MoO_2_(4 h)@Zn is lower than that of MoO_2_(8 h)@Zn and MoO_2_(12 h)@Zn in 1 M ZnSO_4_ with Tw80 (Figure 4c). The reduced *E*_*a*_ of MoO_2_(4 h)@Zn demonstrates that MoO_2_(4 h) coating layer can facilitate rapid Zn^2+^ desolvation and enhance Zn^2+^ transfer and deposition kinetics.^11−13^ Additionally, adding the Tw80 additive reduces *R*_ct_ in MoO_2_@Zn symmetric cells (Figure S19a,d). Moreover, a slight decrease in the *E*_a_ value is observed in both bare Zn and MoO_2_-coated Zn based symmetric cells with the addition of Tw80 in the electrolyte, while the MoO_2_ coating layer can further decrease the *E*_*a*_ value (Figure S20). The combination of the MoO_2_ coating layer and Tw80 additive can lower the activation energy of Zn^2+^ desovlation, enhance the Zn deposition, and thus facilitate the desolvation-reduction process of hydrated Zn ions.^12,13^

The Zn∥Cu half cells are used to investigate the electrochemical reversibility of bare Zn and MoO_2_@Zn in the ZnSO_4_+Tw80 electrolyte. As shown in Figure S21, the initial nucleation overpotential increased from 0.1997 to 0.2696 V after being coated with MoO_2_. A higher nucleation overpotential indicates that the MoO_2_ coating layer reduces the transfer kinetics of Zn^2+^ ions, which is beneficial for refining deposited particles and achieving uniform Zn deposition, consistent with previous reports.^40,62^ The Coulombic efficiency is also examined to evaluate the reversibility of Zn plating/stripping. The CE values of Zn||Cu, MoO_2_@Zn||Cu, and MoO_2_@Zn||MoO_2_@Cu cells using ZnSO_4_+1Tw80 electrolytes are investigated to evaluate the sustainability of the anode behavior by measuring Zn plating/stripping at 1 mA cm^–2^. Figure 4d–f shows the voltage profiles of bare Zn and MoO_2_@Zn anodes. In the initial cycles of the plating/stripping process, a lattice fitting phase (or reshaped Zn coordination) would occur, resulting in relatively low CE.^15^ At different cycles, the voltage profiles of Zn||Cu cells exhibit significant jitter (Figure 4d) because of the nonuniform reshaping of the surface and the formation of “dead Zn” on Cu foil.^15^ Also, MoO_2_@Zn||Cu and MoO_2_@Zn||MoO_2_@Cu cells demonstrate stable voltage profiles at increasing cycle numbers (Figure 4e,f), suggesting an enhancement of surface protection for the plating/stripping process. Therefore, the MoO_2_ coating layer substantially enhances the CE.

A comparison of CE between blank ZnSO_4_ and ZnSO_4_+1Tw80 electrolytes is also conducted to verify the effect of the Tw80 additive on enhancing the reversibility and stability of Zn plating/stripping (Figure 4g,h). The CE of the bare Zn electrode in blank ZnSO_4_ electrolyte quickly decays after 88 cycles, indicating the irregular Zn deposition on Cu foil. On the stable cyclability of bare Zn could be maintained over 118 cycles with the Tw80 additive in ZnSO_4_ electrolyte, which is attributed to the effective regulation of Tw80 on Zn plating/stripping processes. Moreover, while the CE of MoO_2_@Zn cell with ZnSO_4_+1Tw80 electrolyte remains stable over 500 cycles, the cell using blank ZnSO_4_ electrolyte remains relatively stable for 150 cycles and then continuously fluctuates in the following cycles, possibly due to the dendrite formation, byproducts, and other side reactions. As demonstrated by XRD patterns, characteristic peaks of Zn metal can be observed after deposition on Cu foil (Figure S22). The results show that the Zn deposit in ZnSO_4_+1Tw80 electrolyte has a higher intensity ratio of (002) peak to (101) peak, I_(002)_/I_(101)_ than that in blank ZnSO_4_ electrolytes (1.69 and 1.03), which indicates that the ZnSO_4_+1Tw80 electrolyte facilitates a preferential orientation of Zn (002) plane.^63^ Due to this change in texture orientation, the Zn deposited on the electrodes in the ZnSO_4_+1Tw80 electrolyte will be more homogeneous and flatter. Thus, the Tw80 additive can help stabilize the CE.

**Figure 5:**
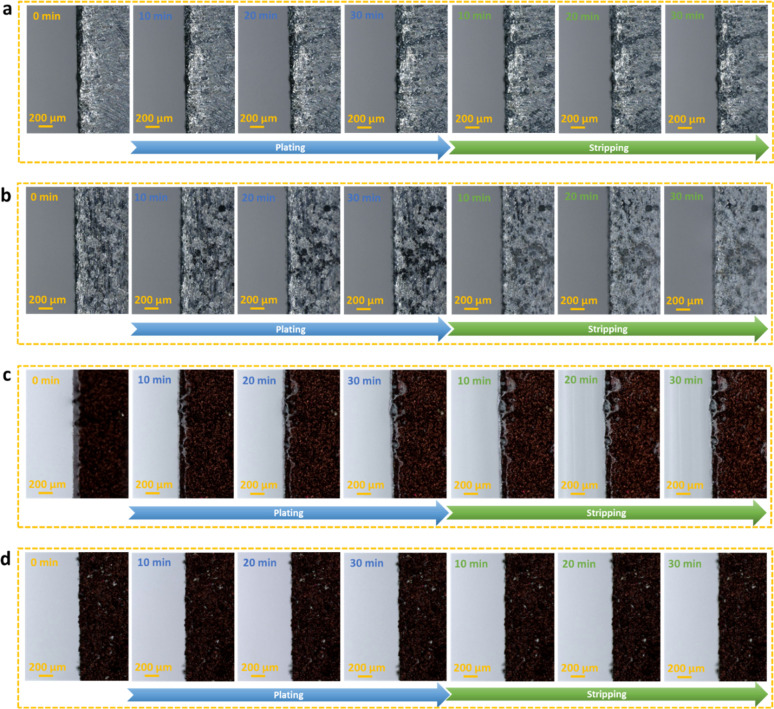
*In situ* optical microscope observation of Zn plating/stripping process for bare Zn using (a) blank ZnSO_4_ electrolyte and (b) ZnSO_4_ containing Tw80 electrolyte at 2 mA cm^–2^ for 30 min. *In situ* optical microscope observation of Zn plating/stripping process for MoO_2_@Zn electrodes using (c) blank ZnSO_4_ electrolyte and (d) ZnSO_4_ containing Tw80 electrolyte at 2 mA cm^–2^ for 30 min.

*In situ* optical microscopy was further employed on transparent cells to investigate the protective effect of the Tw80 additive and MoO_2_ layer on the plating and stripping of the Zn anodes. The fabricated transparent cell is shown in Figure S23. The transparent cells are plated for 30 min and then stripped for 30 min at 2 mA cm^–2^. In a blank ZnSO_4_ electrolyte, numerous Zn nuclei are unevenly distributed on the edge and surface of bare Zn foil after 10 min (Figure 5a and Video S1). It took 30 min for the small protrusion to grow into a prominent Zn dendrite at the exact location. Meanwhile, severe corrosion signs are observed on the Zn surface, and their areas increase as the plating duration increases. In the subsequent stripping process, dendrite growth and corrosion continuously occur on the electrode edge and surface, especially in the area adjacent to the Zn dendrites. According to these observations, the bare Zn electrode using a blank ZnSO_4_ electrolyte exhibits uneven electrodeposition, high corrosion, and weak reversibility. For the bare Zn electrode in a ZnSO_4_+1Tw80 electrolyte, dark spots exist on the surface after 10 min, and their sizes increase as plating proceeds, demonstrating that dendrite growth could occur on the electrode surface (Figure 5b and Video S2). There are also signs of corrosion on the surface of the bare Zn electrode. Besides, the bare Zn electrode surface becomes brighter during the subsequent stripping process, resulting in continuously ununiform Zn dissolution. However, the edge of bare Zn using ZnSO_4_+1Tw80 maintains a smooth and flat shape during the plating/stripping process, indicating that the Tw80 additive can support diminishing dendrite growth. The MoO_2_@Zn electrode is also plated/stripped in blank ZnSO_4_ electrolyte, showing that the surface is almost as smooth as the initial process, and very few dendrites could be observed at the edge after plating/stripping for 30 min (Figure 5c and Video S3). On the contrary, no apparent dendrite growth and corrosion are observed on the MoO_2_@Zn electrode using ZnSO_4_+1Tw80 electrolyte, benefiting from the induced uniform deposition effect of MoO_2_ coating (Figure 5d and Video S4). All of the above results confirm that the combination of MoO_2_ coating with Tw80 additive has synergistic protection on Zn electrodes, effectively homogenizing Zn nucleation and suppressing dendrite growth.

**Figure 6:**
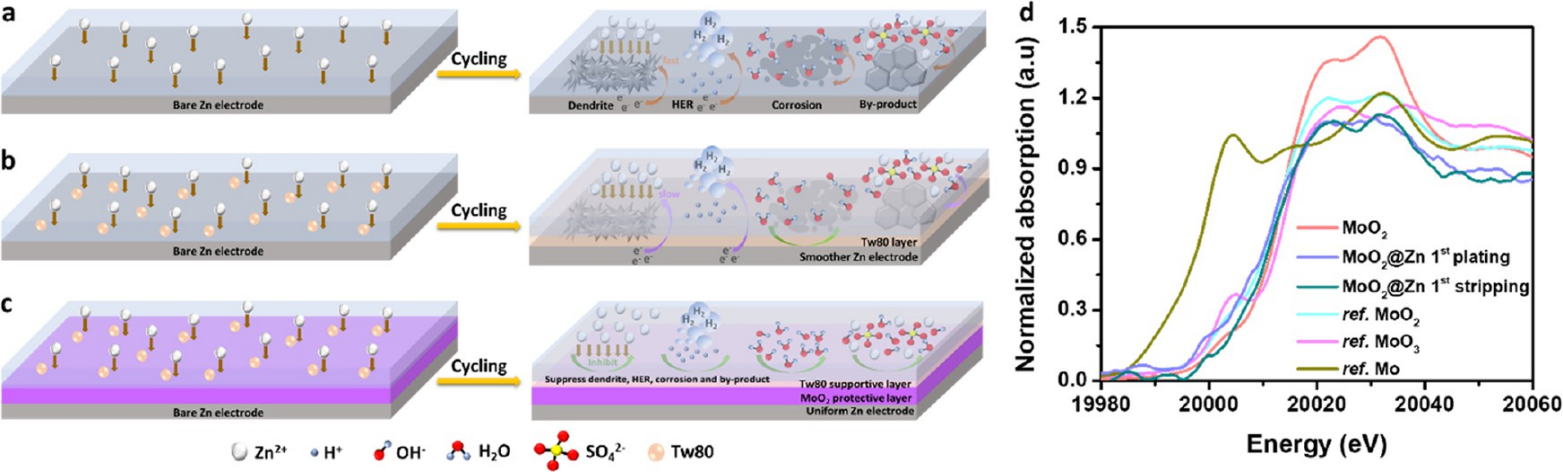
Schematic diagrams of Zn^2+^ ions deposition behaviors in aqueous electrolytes (a) bare Zn in blank ZnSO_4_ electrolyte, (b) bare Zn in blank ZnSO_4_ containing Tw80 electrolyte, and (c) MoO_2_-coated Zn in ZnSO_4_ electrolyte containing Tw80 electrolyte. (d) XANES spectra of MoO_2_@Zn electrodes after plating and stripping in ZnSO_4_+1Tw80 electrolyte.

A detailed mechanism for the contribution of the Tw80 additive and MoO_2_ coating to the regulation of Zn^2+^ ions deposition behavior is presented in Figure 6. It is noticeable that bare Zn foil in an aqueous electrolyte is unstable during the plating/stripping process. According to the “tip effect”, anode surfaces become rough during the initial nucleation process owing to the deposition of Zn^2+^ ions.^64^ Due to the principle of minimizing surface energy and exposed surface area, Zn^2+^ ions adsorbed on the surface tend to diffuse laterally along the surface and generate Zn dendrites. With repeated plating cycles, the Zn^2+^ ions are preferably deposited on the initial dendrites, causing an inhomogeneous electric field distribution. Thus, considerable harmful Zn dendrites are formed on the anode surface (Figure 6a). Since the Zn^2+^ ions are coordinated with H_2_O molecules in ZnSO_4_ aqueous solution, hydride Zn^2+^ ions ([Zn(H_2_O)_6_]^2+^) are the main form of Zn^2+^ ions in the solution, which provides a high desolvation energy of Zn^2+^ ions.^64^ However, [Zn(H_2_O)_6_]^2+^ ions could result in a deprotonation process, creating OH^–^ and H^+^. The produced H^+^ ions cause the HER on the surface of the anode because of its higher redox potential compared with that of Zn deposition. The generated OH^–^ ions incorporate H_2_O molecules to induce corrosion and increase the local pH value by passivating the anode surface to form byproducts (ZSOH). These factors would result in inferior cycling performance and low CEs of ZIBs. Also, the above-mentioned results indicate that only MoO_2_ coatings cannot completely enhance long-term stability for the following reasons: (1) A single MoO_2_ coating layer cannot effectively and durably inhibit corrosion, and (2) it is not an ideally hermetical coating layer that can eliminate electrolyte penetration and corrosion, which may not fully prevent the formation of byproducts and HER.

An effective electrolyte additive for stabilizing the Zn/electrolyte interface is introduced in this work by using Tw80, which is a low-cost and nonionic surfactant. Notably, the Tw80 additive provides promising inhibitors against Zn corrosion in a sulfate solution.^27,28^ First, hydrophilic groups in Tw80 (Figure S24) can form strong hydrogen bonds with water molecules, thereby decreasing the solvation interaction between Zn^2+^ ions and H_2_O molecules and facilitating the desolvation process of hydrated Zn^2+^ ions.^9^ Second, the hydrophobic alkyl chains in Tw80 have a negative inductive effect, leading to the increase of electron density at the oxygen atom along the alkyl chain, thus stabilizing the interaction between Zn and O (on Tw80) and mitigating H_2_O contact with the anode to enhance the protection efficiency.^27^ In the corrosion field, the surfactant like Tw80, as a corrosion inhibitor, makes its hydrophilic portion adsorbed on the metal/metal oxide surface and a second layer (or multiple layers) may form with their hydrophobic tail adjacent to each other in an increased concentration, which can decrease the interfacial tension at the solid–water interface and contact angle and thus enhance the wettability.^58,65^ Based on results of contact angle measurement and EIS under different temperatures (Figures S18–S20), Tw80 can also slightly increase surface hydrophilicity to reduce the desolvation energy of hydrated Zn ions. Thus, Tw80 can act as a corrosion inhibitor to prevent Zn corrosion and side reactions (Figure 6b). Although the Tw80 additive is an effective corrosion inhibitor, dendrites, HER, and byproducts could gradually develop on the bare Zn surface due to the direct contact between the electrolyte and electrode surface. Then, polarization linear sweep voltammetry (LSV) was performed to further examine the effect of the MoO_2_ coating layer and Tw80 additive on suppressing HER. Figure S25 shows the LSV curves of bare Zn in blank ZnSO_4_ electrolyte, and MoO_2_@Zn in ZnSO_4_ electrolyte without Tw80 and with Tw80. The MoO_2_ coating layer can slightly increase the hydrogen evolution overpotential, thereby retarding the HER during cycling in blank ZnSO_4_. In addition, the overpotential apparently increases when the MoO_2_ coating layer is combined with the Tw80 additive, indicating that the combination of MoO_2_@Zn and Tw80 additive is most effective in suppressing HER. Therefore, the combination of the MoO_2_ coating layer and the Tw80 additive can synergistically work to significantly prevent corrosion and side reactions for stabilizing Zn (Figure 6c).

To study the redox behavior of MoO_2_ on protective Zn electrode, Mo K-edge XANES spectra were collected for cycled MoO_2_@Zn electrodes at 5 mA cm^–2^ after the first cycle at both plating/stripping processes (Figure 6d). It is observed that during cycling, the absorption edge of XANES spectra of MoO_2_@Zn electrodes exhibits similar properties except for a slight energy position shift, implying no change in the structure. Specifically, during plating, the edge position shifts to the lower energy suggesting the slightly reduced Mo valence, possibly because MoO_2_ may accommodate a small quantity of Zn^2+^, similar to the reported Li^+^ insertion into the MoO_2_ lattice.^66^ After the stripping, the XANES spectrum demonstrates a slight shift of the absorption edge to a higher energy position closer to the initial state (MoO_2_), implying a recovery of the Mo valence state.^66^ This result indicates a redox reversibility of Mo in the MoO_2_ coating layer during the Zn^2+^ insertion (plating) and extraction (stripping) processes in MoO_2_. This mechanism of Zn^2+^ insertion/extraction into/from MoO_2_ may differ from that of MoO_3_ conversion in LIBs.^66,67^ The Zn-ion storage process for MoO_2_ is similar to that of previously reported MoO_*x*_ for Zn^2+^ storage,^67^ as follows:
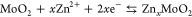
2

The MoO_2_ coating layer with slight Zn^2+^ accommodation capability may help to make the Zn^2+^ concentration field more homogeneously distributed in the vicinity of the Zn anode and avoid a large concentration gradient, which may facilitate the uniform deposition of Zn.

**Figure 7:**
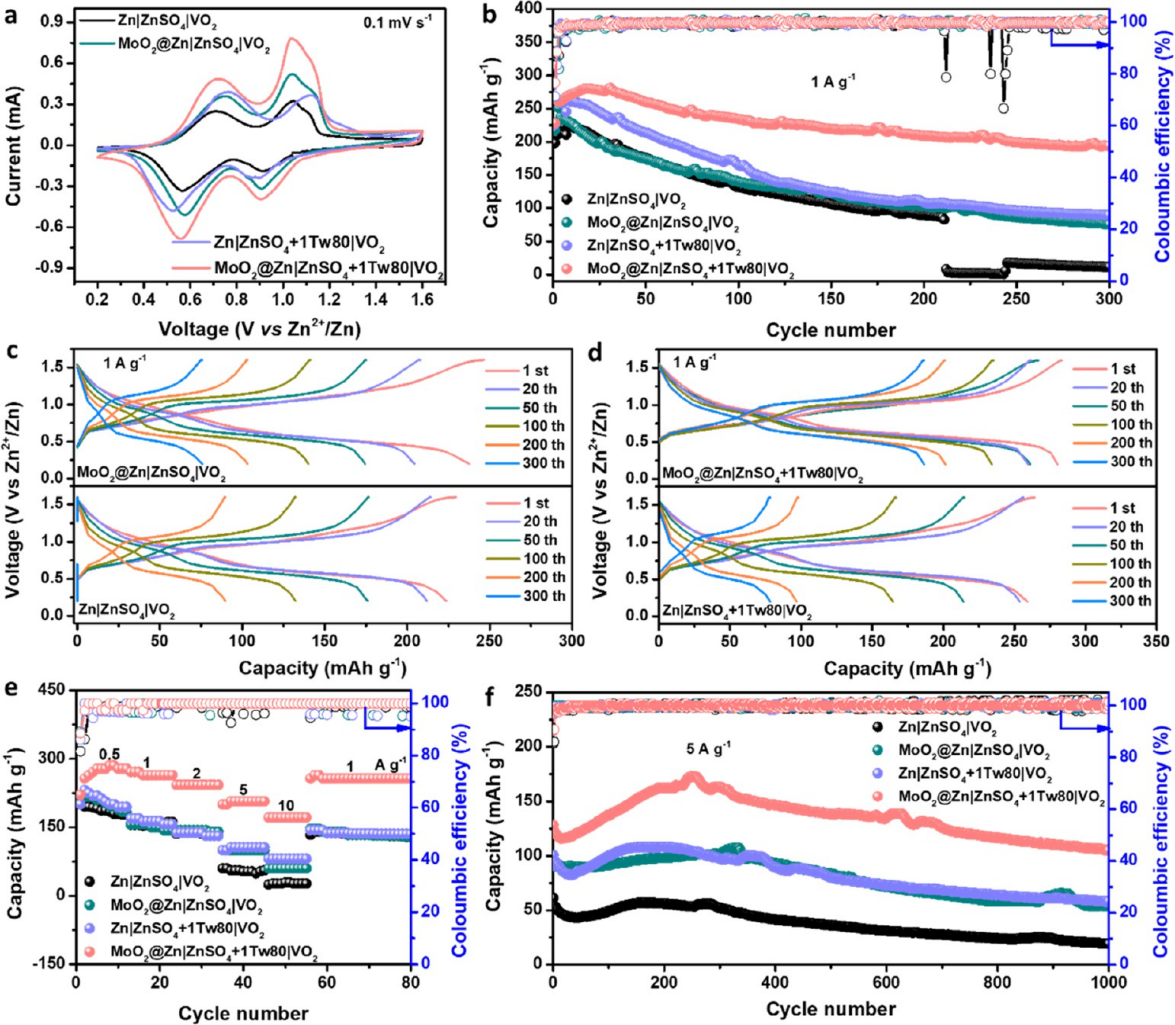
Zn∥VO_2_ and MoO_2_@Zn∥VO_2_ full cells performance with (a) CV curves at a scan rate of 0.1 mV s^–1^. (b) Long-term cycling performance at 1 A g^–1^. GCD curves of different cycles of Zn∥VO_2_ and MoO_2_@Zn∥VO_2_ full cells using (c) ZnSO_4_ and (d) ZnSO_4_+1Tw80 electrolytes at a current density of 1 A g^–1^. (e) Rate performance from 0.5–10 A g^–1^. (f) Cycling stability at a high current density of 5 A g^–1^.

To further investigate the advantages of the MoO_2_ coating layer and Tw80 additive on Zn anode protection, full cells were assembled by using VO_2_ as cathodes. The characterizations of VO_2_ are displayed in Figure S26. A nanorod morphology of VO_2_ is observed in the SEM image. The XRD pattern matches well with the standard card (PDF no. 00–031–1438), confirming the monoclinic phase and purity of the synthesized VO_2_. As shown in Figure 7a, the CV curves at 0.1 mV s^–1^ for the full cell with bare Zn and MoO_2_@Zn anodes in blank ZnSO_4_ and ZnSO_4_+1Tw80 electrolytes are compared within a voltage window of 0.2–1.6 V versus Zn^2+^/Zn. All cells exhibit two pairs of reduction and oxidation peaks, suggesting a two-step Zn^2+^ ion intercalation and deintercalation reactions mechanism during the charge/discharge process, which has been previously reported.^68,69^ The CV curves of the four samples have no considerable difference in the shape, suggesting that the addition of the Tw80 electrolyte additive and MoO_2_ coating layer has no significant effect on the intercalation/deintercalation processes. When Tw80 is added to the Zn∥VO_2_ cell, the peaks are shifted (Figure S27a). However, the CV curves of MoO_2_@Zn∥VO_2_ cells in different electrolytes are almost overlapped, which implies that the redox reaction of the VO_2_ cathode is not affected by the coating layer (Figure S27b). Additionally, MoO_2_@Zn∥VO_2_ cells in different electrolytes demonstrate lower potential separation and higher response current than bare Zn anode (Figure 7a), indicating better reversibility and electrochemical activity, which may be due to the faster kinetics of Zn^2+^ intercalation and weaker side reactions.^70^Figure 7b displays the cycling performance of Zn∥VO_2_ and MoO_2_@Zn∥VO_2_ full cells with blank ZnSO_4_ and ZnSO_4_+1Tw80 electrolytes at a current density of 1 A g^–1^. The cell using the MoO_2_@Zn anode in ZnSO_4_+1Tw80 electrolyte shows the best cycling stability with an initial discharge capacity of 228.3 mAh g^–1^ and capacity retention of 85.2% after 300 cycles, while the capacity retention decreases rapidly to 39.9% after cycling for the cell with a bare Zn anode. In contrast, the capacity of Zn∥VO_2_ cell in blank ZnSO_4_ drops to <10 mAh g^–1^ after 210 cycles and the MoO_2_@Zn∥VO_2_ cell exhibits a capacity retention of 34.8% after 300 cycles. The galvanostatic charge/discharge profiles (GCD) of full cells are shown in Figures 7c,d and S28. Likewise, the full cells with ZnSO_4_ and ZnSO_4_+1Tw80 electrolytes display a similar charge/discharge behavior. The cells using ZnSO_4_+1Tw80 electrolyte can sustain a much longer cycle lifespan and much better stability than those using a blank ZnSO_4_ electrolyte. The results demonstrate that the combination of the MoO_2_@Zn anode and Tw80 electrolyte additive could prolong the lifetime of Zn∥VO_2_ full cells.

Additionally, the rate performance of the full cell with the MoO_2_@Zn anode is superior to that of the cell with the bare Zn anode in different electrolytes as the current density increases from 1 to 10 A g^–1^ (Figure 7c). Even at a high current density of 10 A g^–1^, the MoO_2_@Zn∥ZnSO_4_+1Tw80∥VO_2_ full cell demonstrates a large initial capacity of 171.4 mAh g^–1^, while Zn∥ ZnSO_4_+1Tw80∥VO_2_ and MoO_2_@Zn∥ZnSO_4_∥VO_2_ show the capacity below 100 mAh g^–1^ and the capacity of Zn∥ZnSO_4_∥VO_2_ is less than 50 mAh g^–1^. Upon long-term cycling at a current density of 5 A g^–1^, the MoO_2_@Zn∥ZnSO_4_+1Tw80∥VO_2_ cell maintains outstanding performance with a high retained capacity of 105.6 mAh g^–1^ and superior capacity retention of 82.4% after 1000 cycles (Figure 7d). Conversely, the specific capacity of the Zn∥ZnSO_4_+1Tw80∥VO_2_ cell is reduced to 57.7 mAh g^–1^, corresponding to a capacity retention of 57.3%. At 5 A g^–1^, Zn∥VO_2_ and MoO_2_@Zn∥VO_2_ cells using blank ZnSO_4_ display the same electrochemical performance trend for 1000 cycles. However, the capacity retention of these full cells drops quickly to 59.3% and 31.2%, respectively. Significantly increasing the current density to 10 A g^–1^, the MoO_2_@Zn∥VO_2_ cell in the Tw80-assisted electrolyte system maintains an exceptionally stable performance (Figure S29). Overall, the outstanding electrochemical performances of the Zn anodes can be attributed to the effective inhibition of corrosion, HER, and dendrite formation in the presence of the MoO_2_ layer incorporating Tw80 electrolyte additive.

## 4 Conclusion

In summary, this work proposed a new approach to developing a highly stable Zn anode by combining a surface coating layer and an electrolyte additive. The combination of MoO_2_ coating layer and the Tw80 additive can synergistically work to significantly prevent corrosion and side reactions. It can also reduce the desolvation energy of hydrated Zn ions during cycling, promoting uniform Zn^2+^ stripping/plating and improving the cell electrochemical performances. Benefiting from the dual synergistic effects of the MoO_2_ coating layer and Tw80 additive, the MoO_2_@Zn anode exhibits excellent electrochemical stability in a 1 M ZnSO_4_ electrolyte. The MoO_2_@Zn anode shows ultralong cycling stability in symmetric cells over 6000 h at 1 mA cm^–2^ for 1 mAh cm^–2^. The symmetric cells also show long-term stability over 705 h, even at a high current density of 5 mA cm^–2^ and areal capacity of 5 mAh cm^–2^. Especially, the full cells exhibit a high capacity of 105.6 mAh g^–1^ after 1000 cycles with outstanding rate performance (171.4 mAh g^–1^ at 10 A g^–1^). This work provides a method of combining the artificial coating layer and electrolyte additive to stabilize Zn for high-performance aqueous ZIBs.

## Supporting Information Available

Experimental section; SEM images of synthesized MoO_3_ and bare Zn electrode; XPS survey spectra of MoO_3_ and MoO_2_; voltage profiles of bare Zn and MoO_2_@Zn symmetric cells at different cycles; cycling performance of bare Zn and MoO_2_@Zn symmetric cells under high current density, MoO_2_@Zn symmetric cells with different electrolytes, MoO_3_@Zn and different MoO_2_@Zn symmetric cells; characterization of MoO_2_@Zn electrodes after cycling with different electrolytes (SEM images and XRD results); characterization of different MoO_2_ (SEM images and XRD, EPR results); contact angles of bare Zn and MoO_2_@Zn with different electrolytes; optical images of MoO_2_@Zn soaking in different electrolytes and corresponding XRD patterns and SEM images; EIS spectra at different temperatures, calculated activation energy, Tafel plots, CA curves of different electrodes in different electrolytes; XRD results of bare Cu foils after Zn deposition in different electrolytes and corresponding peak intensity ratio; digital image of transparent cell using for optical *in situ* microscope observation; structure of Tween 80; characterization of the VO_2_ cathode (SEM image and XRD result) and corresponding electrochemical performance of Zn∥VO_2_ and MoO_2_@Zn∥VO_2_ full cells in different electrolytes (PDF)
Video S1: *In situ* optical microscope testing of bare Zn electrode in blank ZnSO_4_ electrolyte (MP4)
Video S2: *In situ* optical microscope testing of MoO_2_@Zn electrode in blank ZnSO_4_ electrolyte (MP4)
Video S3: *In situ* optical microscope testing of bare Zn electrode in ZnSO_4_-containing Tween 80 electrolyte (MP4)
Video S4: *In situ* optical microscope testing of MoO_2_@Zn electrode in ZnSO_4_-containing Tween 80 electrolyte (MP4)
