# Supplementary material for: Synergistically Stabilizing Zinc Anodes by Molybdenum Dioxide Coating and Tween 80 Electrolyte Additive for High-Performance Aqueous Zinc-Ion Batteries
Source: ACS Appl Mater Interfaces. 2023 Nov 21;15(48):55570–86. doi: 10.1021/acsami.3c08474 (PMC10711716; doi:10.1021/acsami.3c08474)
Supplement: Supplementary file 1 — am3c08474_si_001.pdf [file am3c08474_si_001.pdf]

Supporting Information

Synergistically Stabilizing Zinc Anodes by Molybdenum Dioxide Coating and Tween 80 Electrolyte Additive for High-Performance Aqueous Zinc-Ion Batteries

Nhat Anh Thieu[1,+], Wei Li[1,+,*], Xiujuan Chen[1], Qingyuan Li[1], Qingsong Wang[2], Murugesan Velayutham[3,4], Zane M. Grady[5], Xuemei Li[6], Wenyuan Li[6], Valery V. Khramtsov[3,4], David M. Reed[5], Xiaolin Li[5,*], and Xingbo Liu[1,*]

[1]Department of Mechanical and Aerospace Engineering, Benjamin M. Statler College of Engineering and Mineral Resources, West Virginia University, Morgantown, WV 26506, USA

[2]Bavarian Center for Battery Technology (BayBatt), Department of Chemistry, University of Bayreuth, Universitätsstrasse 30, 95447 Bayreuth, Germany

[3]In Vivo Multifunctional Magnetic Resonance Center, Robert C. Byrd Health Sciences Center, West Virginia University, Morgantown, WV 26506, USA

[4]Department of Biochemistry and Molecular Medicine, School of Medicine, West Virginia University, Morgantown, WV 26506, USA

[5]Energy and Environmental Directorate, Pacific Northwest National Laboratory, Richland, WA 99352, USA

[6]Department of Chemical and Biomedical Engineering, Benjamin M. Statler College of Engineering and Mineral Resources, West Virginia University, Morgantown, WV 26506, USA

[+]These authors (N.A.T. and W.L.) contributed equally to this work.

*Authors to whom correspondence should be addressed.

E-mail: wei.li@mail.wvu.edu (W. L), xiaolin.li@pnnl.gov (X. Li), xingbo.liu@mail.wvu.edu (X. Liu)

1. Experimental Section

1.1. Materials

Zinc foils (Zn, 0.1 mm) were purchased from Goodfellow. Carbon papers were purchased from Fuel Cell Store. Ammonium molybdate tetrahydrate ($(NH_4)_6Mo_7O_{24}\cdot 4H_2O$), ethylene glycol (EG), nitric acid 70% (HNO_3), and Tween 80 (average M_w=1310) were obtained from Sigma Aldrich. Zinc sulfate heptahydrate ($ZnSO_4\cdot 7H_2O$) was obtained from Acros Organics. Vanadium oxide (V_2O_5) and ethanol were purchased from Alfa Aesar. Carboxymethyl cellulose (CMC) and conductive carbon black (super P) were obtained from MTI Corporation. Glass microfiber (GF/B) was obtained from Whatman PLC. Deionized (DI) water was used through the experiment.

1.2. Synthesis of MoO_3 nanobelts

Firstly, MoO_3 nanobelts were synthesized by a hydrothermal method.[1,2] 2 g of $(NH_4)_6Mo_7O_{24}\cdot 4H_2O$ was dissolved in 66 mL of DI water and 12 mL of HNO_3 by magnetic stirring for 20 min. The mixture was then transferred to a 100-mL Teflon-line autoclave and placed in an oven for the hydrothermal reaction at 180 °C for 24 h. The obtained solution was centrifuged at 8000 rpm for 3 min and washed with DI water several times. After drying in a vacuum oven at 60 °C for 24 h, MoO_3 was prepared.

1.3. Material characterization

A scanning electron microscope (SEM, JEOL JSM–7600F) equipped with energy-dispersive X-ray spectroscopy (EDS) was used to characterize samples' morphology and elemental composition. X-ray diffraction (XRD, PANalytical X′ Pert Pro) was conducted to characterize the phase structure of samples at 45 kV and 40 mA with Cu Kα radiation. X-ray photoelectron spectroscopy (XPS, PHI 5000 Versa Probe) determined the surface chemical states with a monochromatic Al Kα source. X-ray absorption spectroscopy (XAS) measurements at Mo K-edge were performed at beamline P65 of PETRA III extension of DESY (Hamburg, Germany). The monochromatic beam was produced through a water-cooled double crystal monochromator (Si 111), and the beam was focused by two Rh-coated plane mirrors.[3] XAS spectra between 19850 and 21000 eV were collected in transmission mode. Continuous wave electron paramagnetic resonance (EPR) spectra were recorded on a Bruker ELEXSYS E580 EPR spectrometer (Bruker BioSciences, Billerica, MA) operating at X-band with a 100 kHz modulation frequency, as described previously.[4,5] Powder samples were loaded into an EPR quartz tube (OD 4 mm and ID

3 mm, Wilmad LabGlass, Vineland, NJ, USA) and placed inside the finger Dewar containing liquid nitrogen. The finger Dewar was positioned inside the EPR resonator, and the sample position was adjusted to the center of the sensitive part of the resonator. The EPR measurements were performed at 77 K (liquid nitrogen temperature). EPR data were collected using the Bruker Xepr program. The following instrument settings were used: microwave frequency, 9.554 GHz; magnetic field range, 1000 G; microwave power, 1.5 mW; modulation amplitude, 1 G; receiver gain, 60 dB; conversion time, 40 ms, number of points, 4096; scan time, 164 s; number of scans, 5. EPR spectra were plotted using GraphPad Prism version 9 software/program (GraphPad Software Inc., San Diego, USA). The contact angle measurements were collected using the Keyence VHX-7000 microscope, and the angles were analyzed using Fiji-ImageJ2. For measuring the electrical conductivity, 500 mg MoO_2 and MoO_3 powders were pressed and heated to prepare pellets. The electrical conductivity of the pellets was determined by recording the voltage response while applying a constant current. The electrical conductivity (σ) can be calculated as follows.

$$\sigma = \frac{t}{A} \times \frac{I}{U} \qquad (Eq.\ S1)$$

where t is the thickness of the pellet, A is the circular pellet area, I is the applied current, and U is the measured voltage response.

2. Results

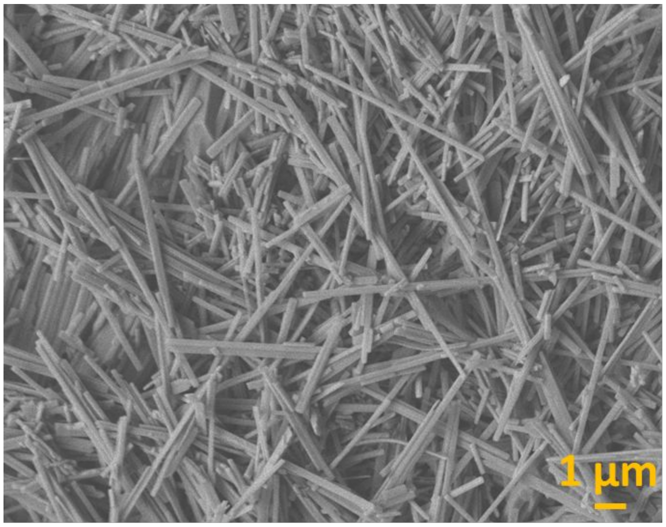

Figure S1. Morphology of the synthesized MoO_3

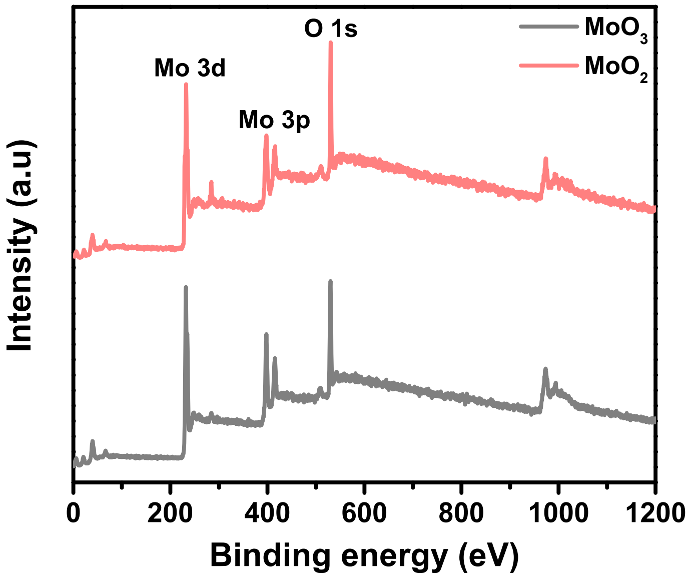

Figure S2. XPS survey spectrum of MoO_3 and MoO_2

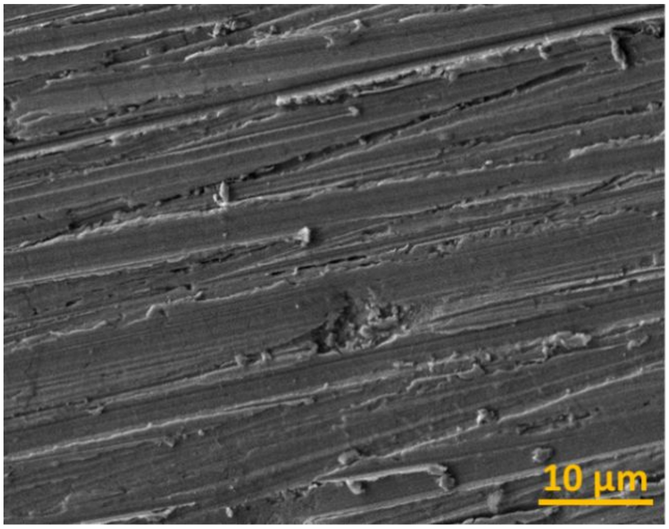

Figure S3. Surface morphology of bare Zn.

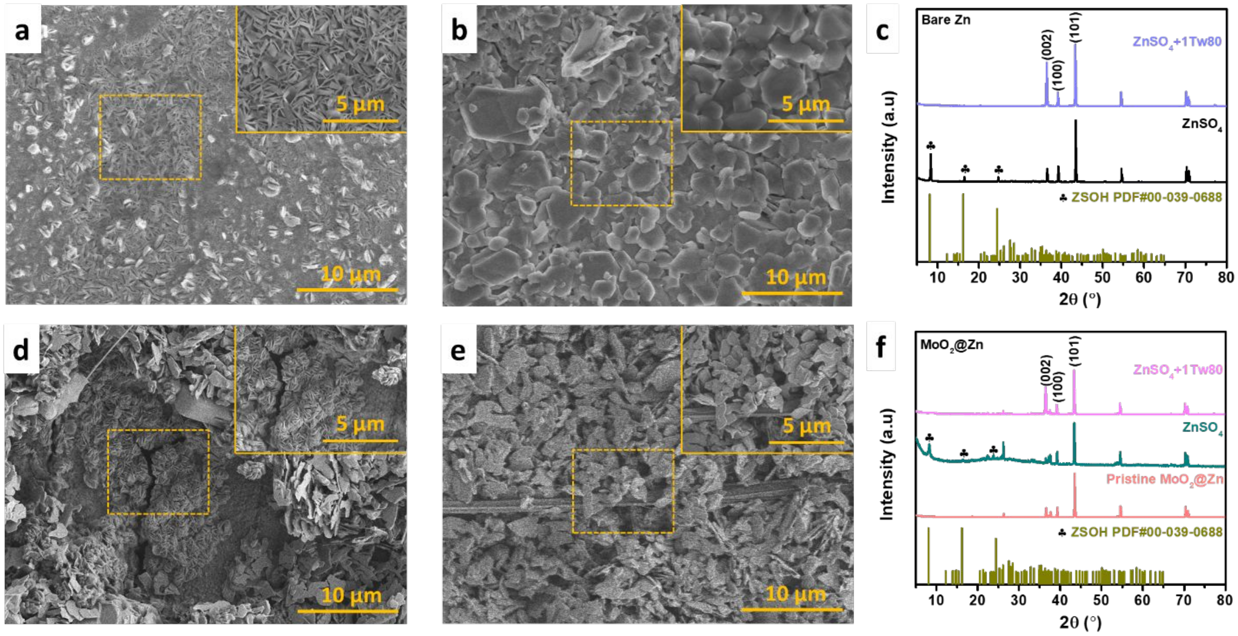

Figure S4. SEM images of bare Zn electrode after cycling at 1 mA cm^{-2}–1 mAh cm^{-2} for 50 cycles in (a) blank $ZnSO_4$ and (b) $ZnSO_4$+1Tw80 electrolytes with (c) corresponding XRD patterns. SEM images of MoO_2@Zn electrode after cycling at 1 mA cm^{-2}–1 mAh cm^{-2} for 50 cycles in (d) blank $ZnSO_4$ and (e) $ZnSO_4$+1Tw80 electrolytes with (f) corresponding XRD patterns.

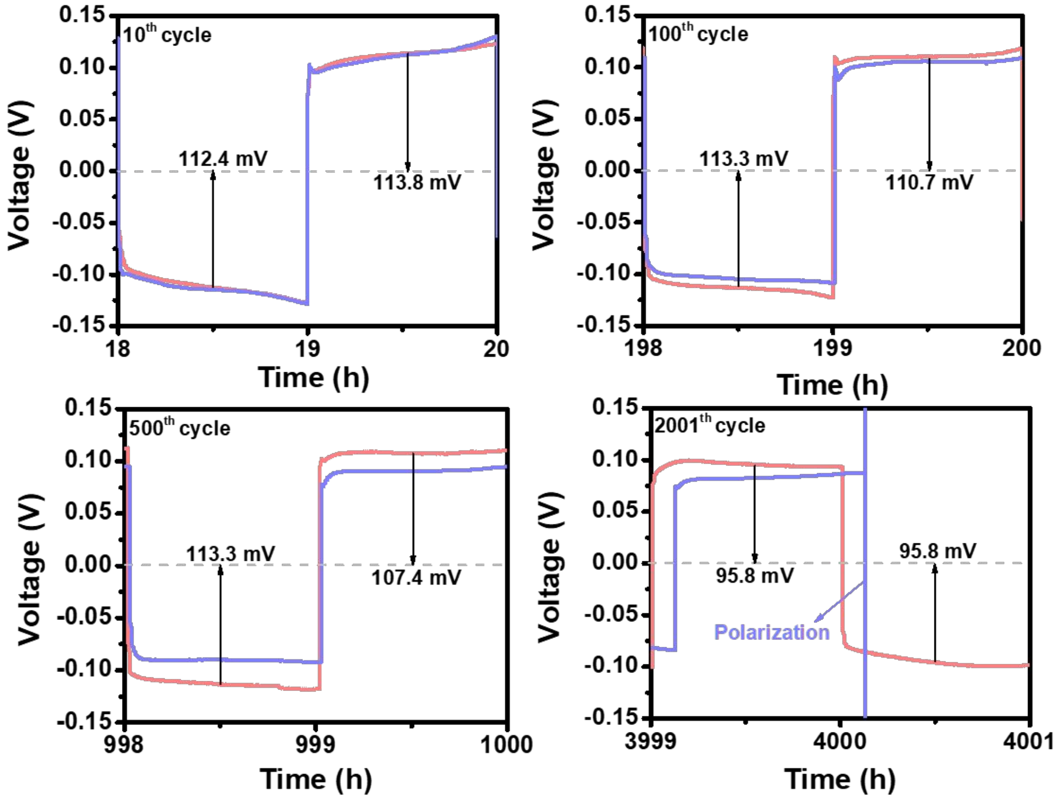

Figure S5. Magnified voltage profiles of bare Zn (purple line) and MoO_2@Zn (red line) symmetric cells with $ZnSO_4$+1Tw80 electrolyte at different cycles.

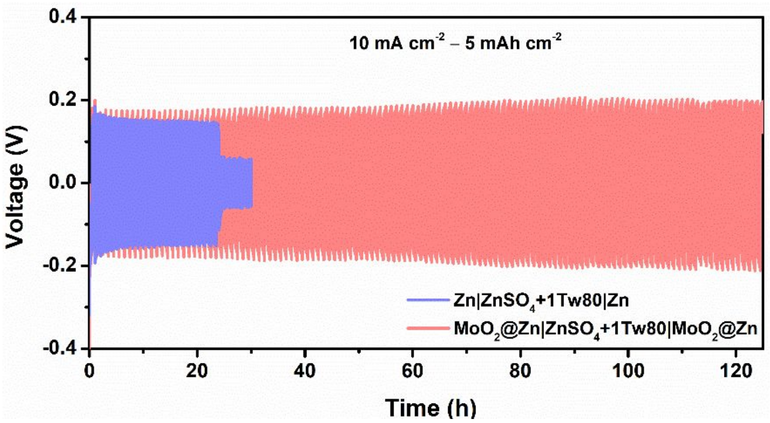

Figure S6. Cycling performance of bare Zn and MoO_2@Zn symmetric cells using $ZnSO_4$+1Tw80 electrolyte at 10 mA cm^{-2}–5 mAh cm^{-2}.

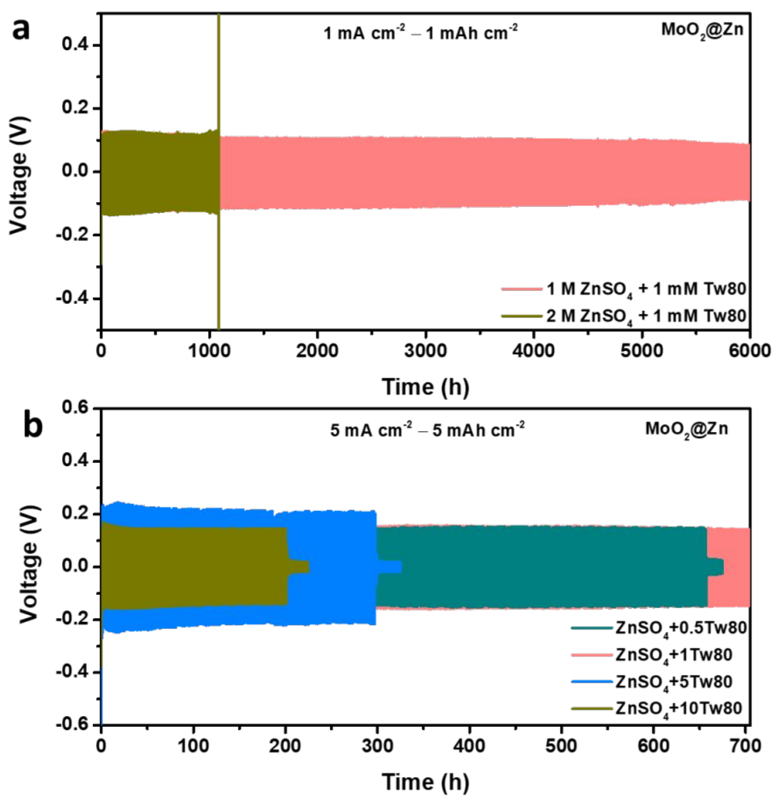

Figure S7. Cycling performance of MoO_2 symmetric cells with (a) different $ZnSO_4$ concentration at fixed 1 mM Tw80 and (b) different Tw80 concentration at fixed 1M $ZnSO_4$.

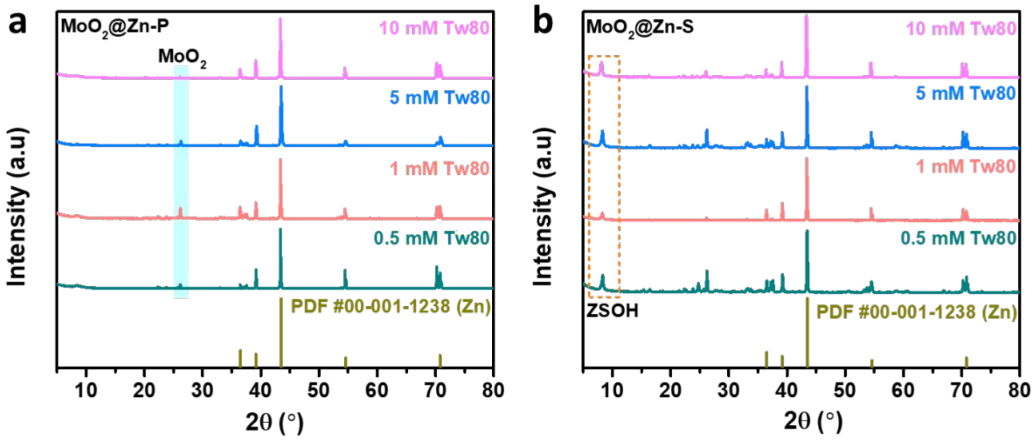

Figure S8. XRD patterns of MoO_2@Zn symmetric cells using different Tw80 concentration at 5 mA cm^{-2}–5 mAh cm^{-2} after 100 cycles at (a) plating state and (b) stripping state.

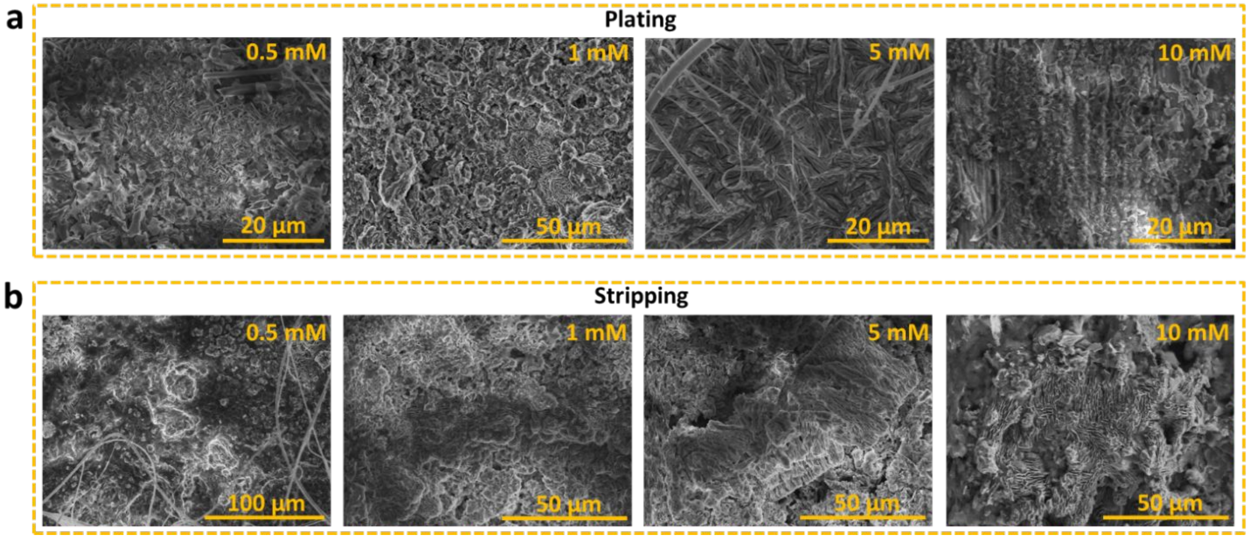

Figure S9. SEM images of MoO_2@Zn symmetric cells using different Tw80 concentration at 5 mA cm^{-2}–5 mAh cm^{-2} after 100 cycles at (a) plating state and (b) stripping state.

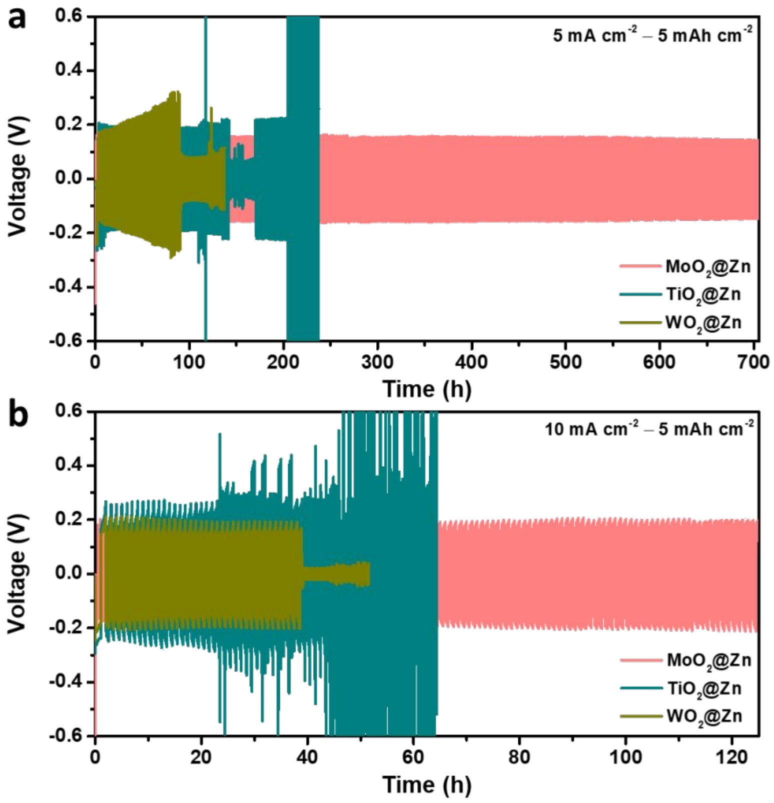

Figure S10. Cycling performance of TiO_2@Zn, WO_2@Zn, and MoO_2@Zn symmetric cells using $ZnSO_4$+1Tw80 electrolytes at (a) 5 mA cm^{-2}, and (b) 10 mA cm^{-2} with a fixed areal capacity of 5 mAh cm^{-2}.

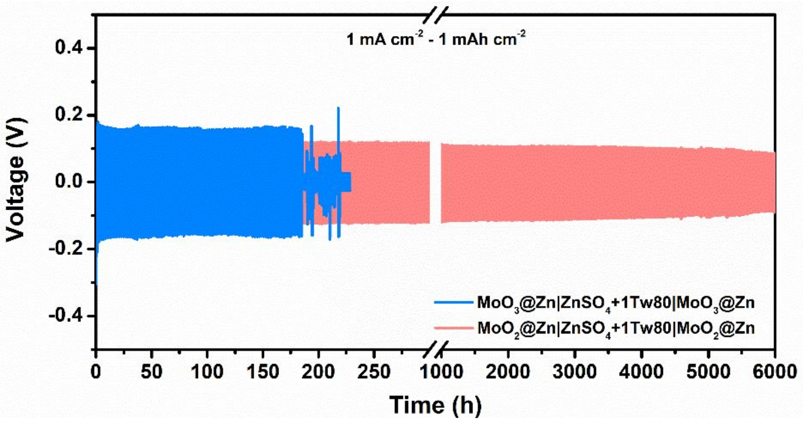

Figure S11. Cycling performance of MoO_3 and MoO_2 coated Zn symmetric cells.

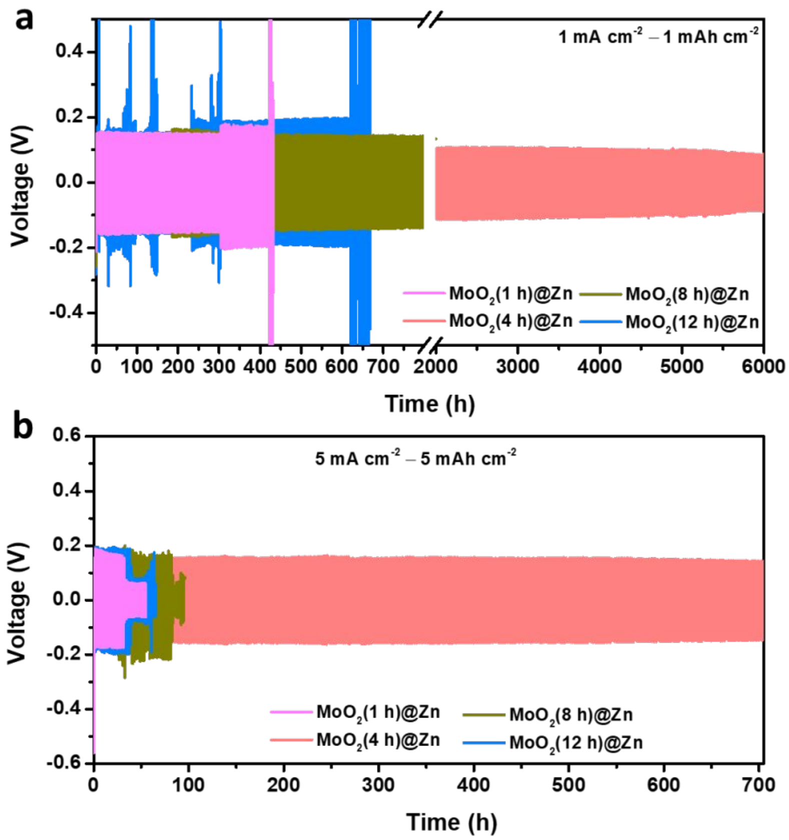

Figure S12. Cycling performance of different MoO_2 coated Zn symmetric cells at different current densities and areal capacities of (a) 1 mA cm^{-2}–1 mAh cm^{-2} and (b) 5 mA cm^{-2}–5 mAh cm^{-2}.

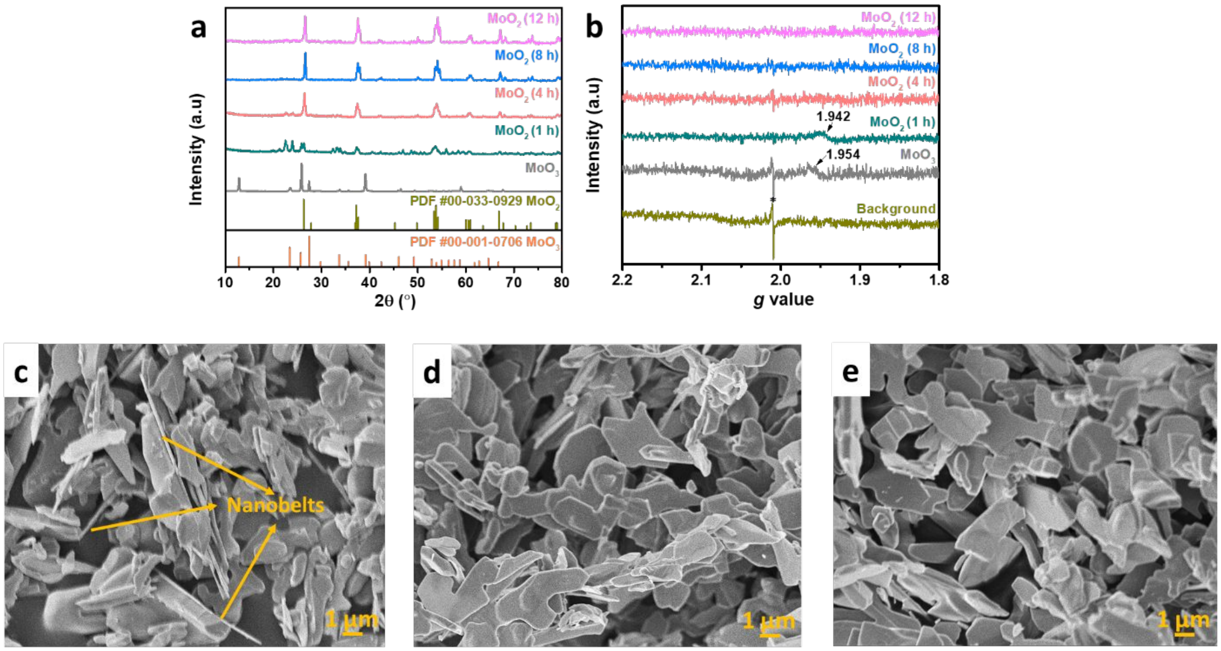

Figure S13. (a) XRD pattern, and (b) EPR spectra of MoO_3 and different MoO_2. The peak labeled by an asterisk was observed in an empty capillary as the background signal. SEM images of MoO_2 at different thermal reduction times (c) 1 h, (d) 8 h, and (e) 12 h.

After reducing for 1 h, the obtained MoO_2 is mostly nanoplates, although some nanobelts could be observed. However, as the reducing time increases, all MoO_2 exhibit the plates-like morphology and are stacked more tightly without space around nanoplates, increasing agglomeration.

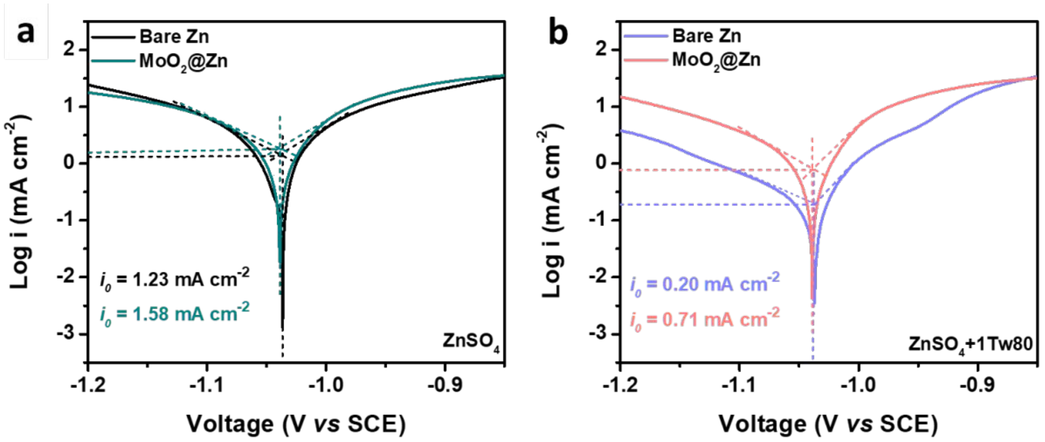

Figure S14. Comparison of Tafel plots of bare Zn and MoO_2@Zn electrodes in (a) $ZnSO_4$ and (b) $ZnSO_4$+1Tw80 electrolytes.

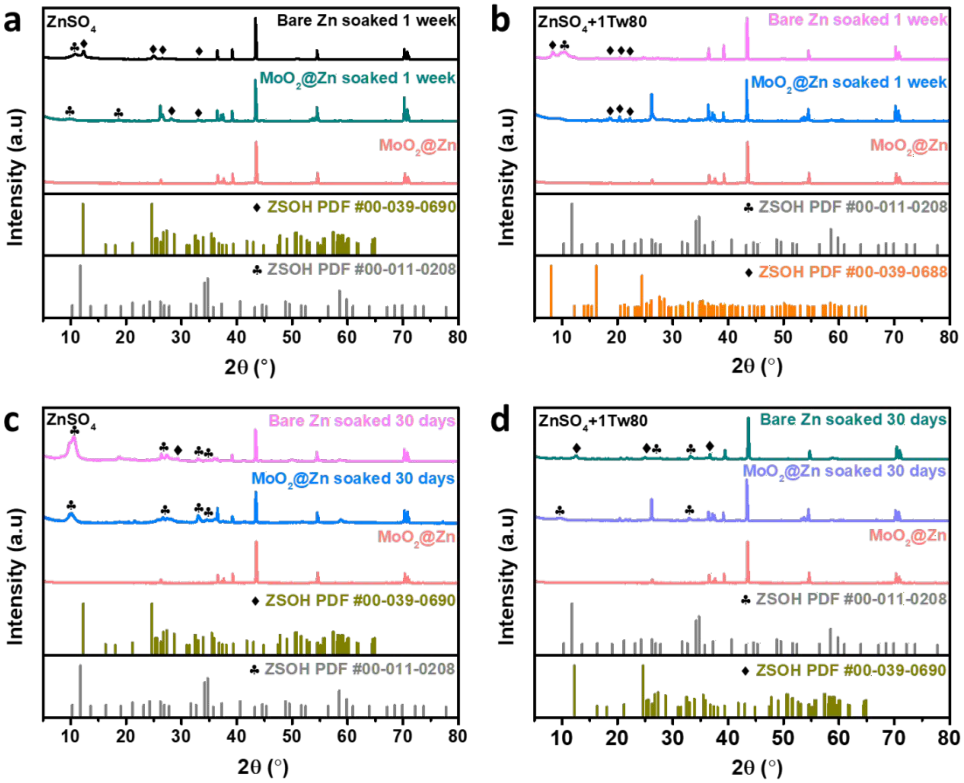

Figure S15. XRD patterns after soaking for 1 week of bare Zn and MoO_2@Zn in (a) $ZnSO_4$ and (b) $ZnSO_4$+1Tw80 electrolytes. XRD patterns after soaking for 30 days of bare Zn and MoO_2@Zn in (c) $ZnSO_4$ and (d) $ZnSO_4$+1Tw80 electrolytes.

As shown in **Figure S15**, XRD patterns revealed that many ZSOH by-products could be found on bare Zn and MoO_2@Zn electrodes after a short duration (1 week) and a long duration (30 days) in $ZnSO_4$ electrolyte. At the same time, few ZSOH could be found on both electrodes after soaking in $ZnSO_4$+Tw80 electrolyte.

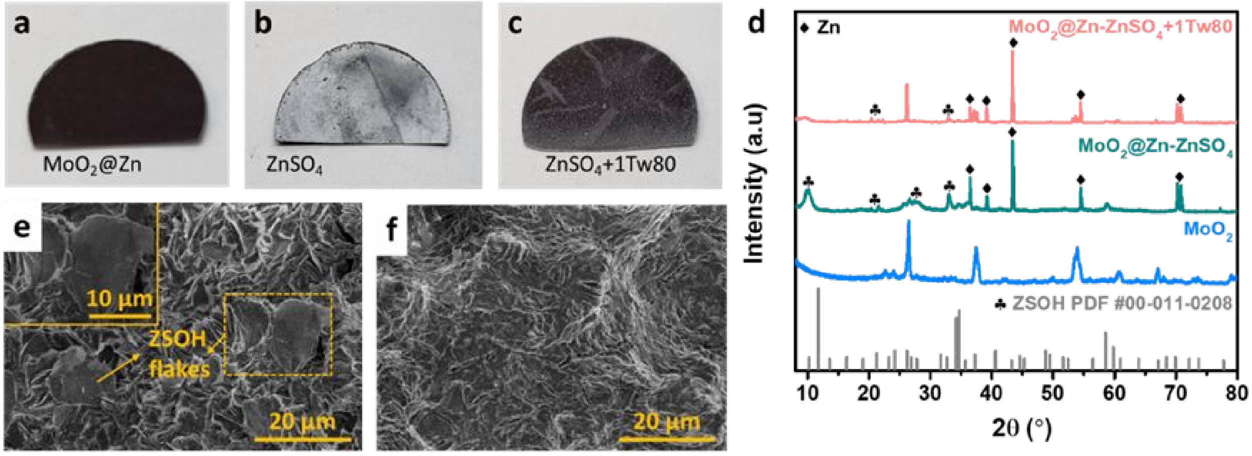

Figure S16. Optical images of (a) pristine MoO_2@Zn and MoO_2@Zn foils soaked in (b) $ZnSO_4$, and (c) $ZnSO_4$+1Tw80 electrolytes after 30 days. (d) Corresponding XRD patterns and SEM images of MoO_2@Zn foils soaked in (e) $ZnSO_4$ with yellow arrows and dashed line boxes labeling the ZSOH flakes with MoO_2 and its high magnification showing the morphology of ZSOH (left inset), and (f) $ZnSO_4$+1Tw80 electrolytes.

MoO_2@Zn plates soaked in $ZnSO_4$-based electrolytes with and without Tw80 are characterized to observe the surface morphology and phase changes. The surface of the MoO_2@Zn plate is seriously corroded after 30 days of soaking in a blank $ZnSO_4$ electrolyte by changing the color from dark brown to white (**Figure S16**a,b). While in the $ZnSO_4$+1Tw80 electrolyte, the color of the MoO_2 coating layer slightly changes to light brown (**Figure S16**c). Further, the XRD patterns and SEM images reveal that after immersion in $ZnSO_4$ electrolyte, lots of ZSOH by-products form on the entire surface of Zn by the growth of noticeable flakes (**Figure S16**d,e). On the other hand, the MoO_2@Zn plate immersed in $ZnSO_4$+1Tw80 shows no visible morphological changes and corrosion by-products (**Figure S16**d,f), indicating an excellent ability to prevent corrosion of the Tw80 additive.

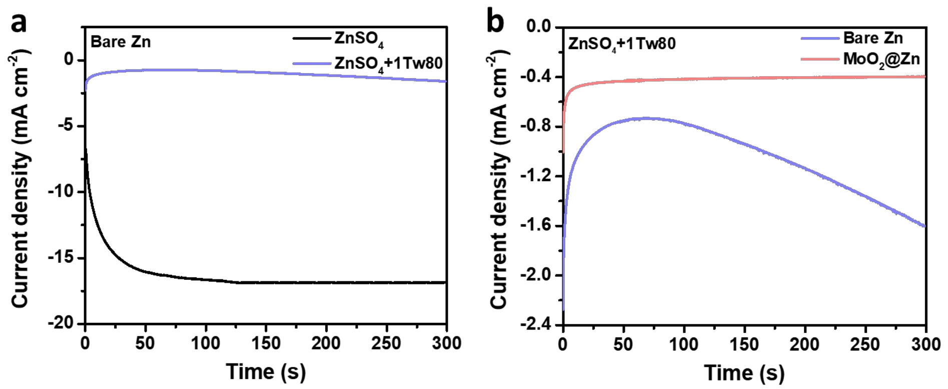

Figure S17. (a) CA curves of bare Zn electrode in different electrolytes. (b) Enlarged view of CA curves of bare Zn and MoO_2@Zn electrodes in $ZnSO_4$+1Tw80 electrolyte.

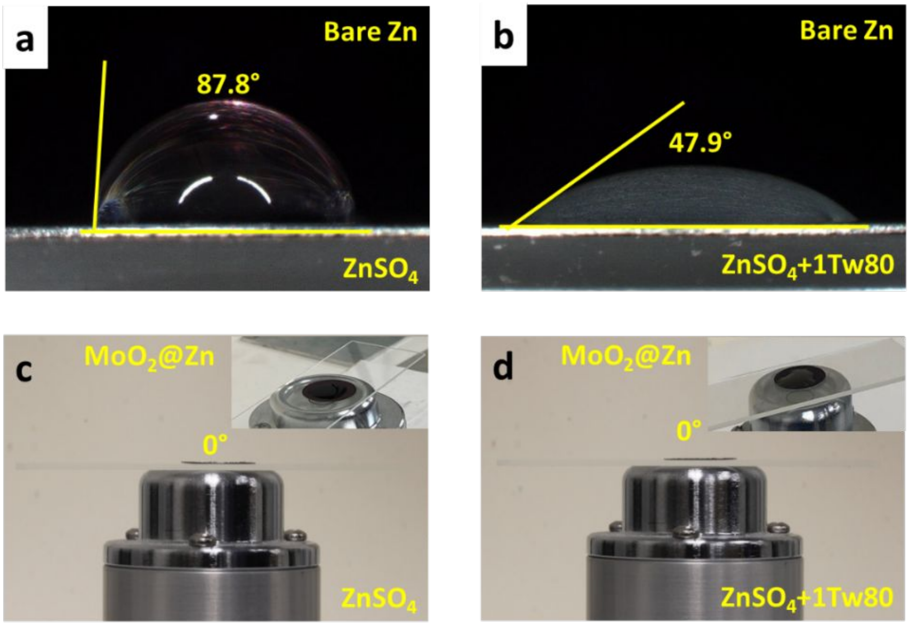

Figure S18. Contact angle measurements for (a) bare Zn in blank $ZnSO_4$ electrolyte, (b) bare Zn in $ZnSO_4$+1Tw80 electrolyte, (c) MoO_2@Zn in blank $ZnSO_4$ electrolyte, and (d) MoO_2@Zn in $ZnSO_4$+1Tw80 electrolyte.

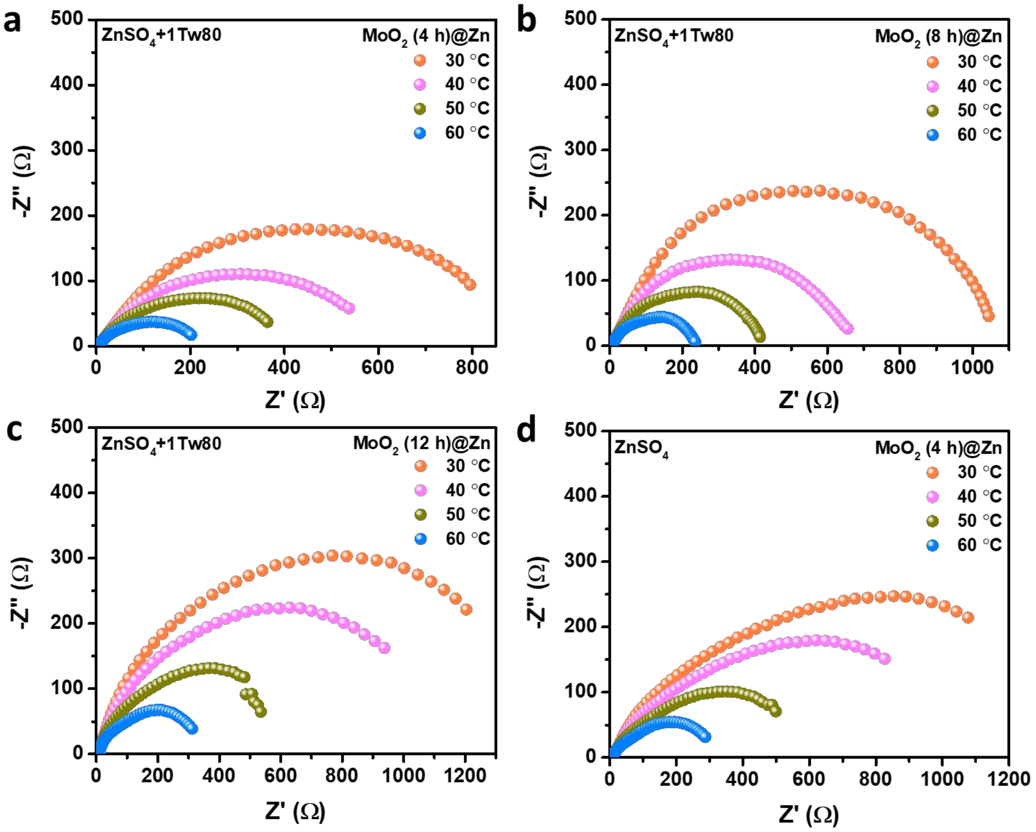

Figure S19. EIS spectra at different temperatures of symmetric cells with (a) MoO_2(4 h)@Zn, (b) MoO_2(8 h)@Zn, and (c) MoO_2(12 h)@Zn in $ZnSO_4$+1Tw80 electrolyte. (d) EIS spectra at different temperatures of MoO_2(4 h)@Zn symmetric cell in blank $ZnSO_4$ electrolyte.

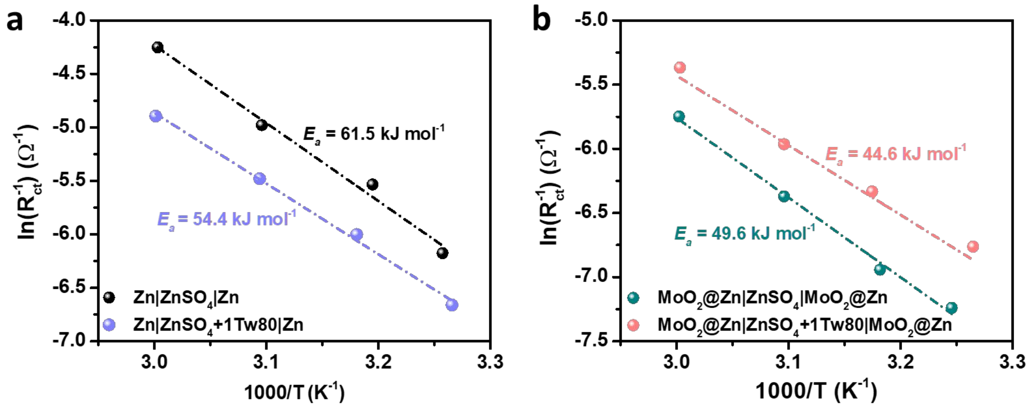

Figure S20. Calculated activation energy of (a) bare Zn and (b) MoO_2@Zn in different electrolytes.

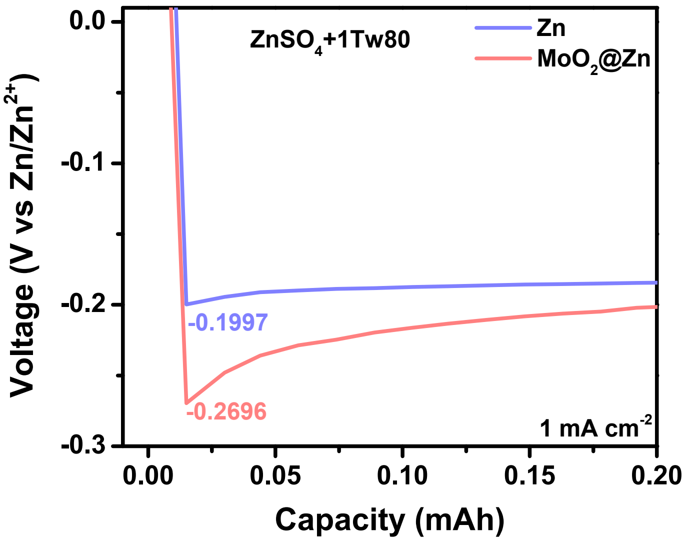

Figure S21. Nucleation overpotential of bare Zn and MoO_2@Zn.

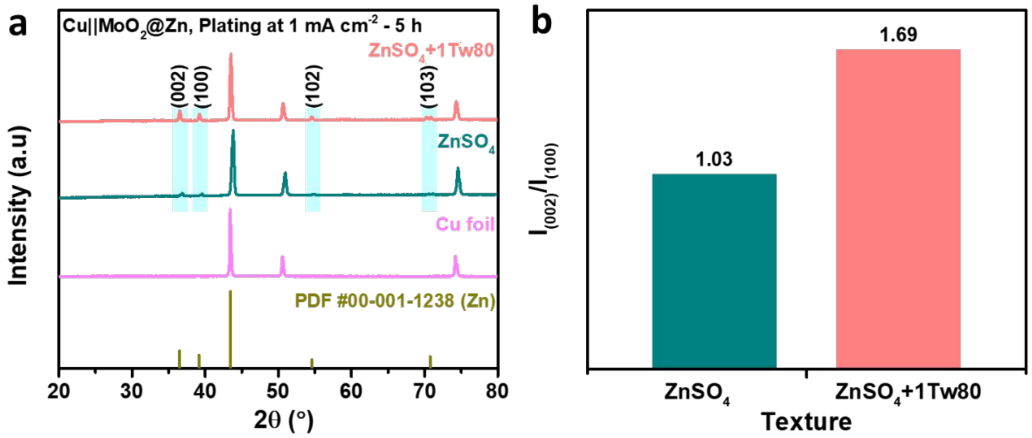

Figure S22. (a) XRD patterns of bare copper foil and that after the Zn deposition for 5 h at 1 mA cm^{-2} –1mAh cm^{-2} with and without Tw80 additive, and (b) Corresponding peak intensity ratio of $I_{(002)}/I_{(100)}$.

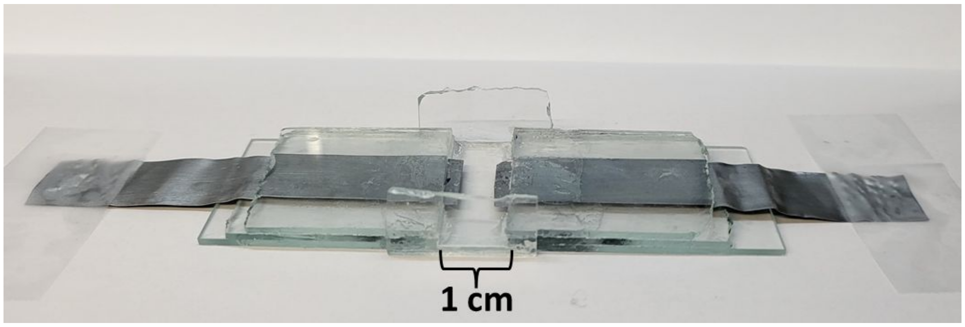

Figure S23. The transparent cell used for optical *in situ* microscope observation.

The transparent cell consists of two pieces of Zn or MoO_2@Zn (1 cm × 5 cm), which are attached to eight pieces of glass slides by using super glue. The distance between two sides of glass slides is 1 cm (as shown in the Figure), where the electrolyte will be contained. The exposed area of each electrode is maintained at 0.2 cm × 1 cm.

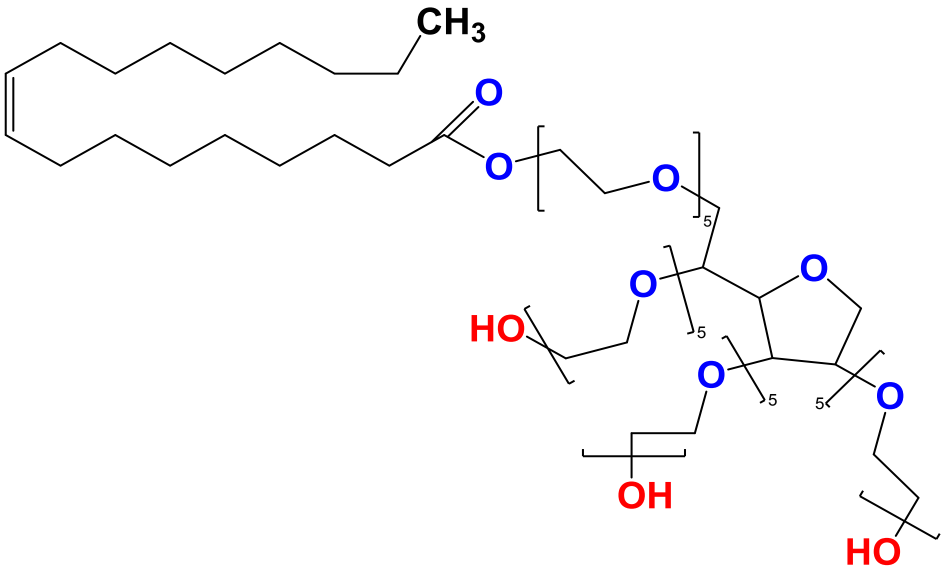

Figure S24. Structure of Tween 80. The structure of Tween 80 consists of an alkyl chain (hydrophobic), and −OH groups (hydrophilic).

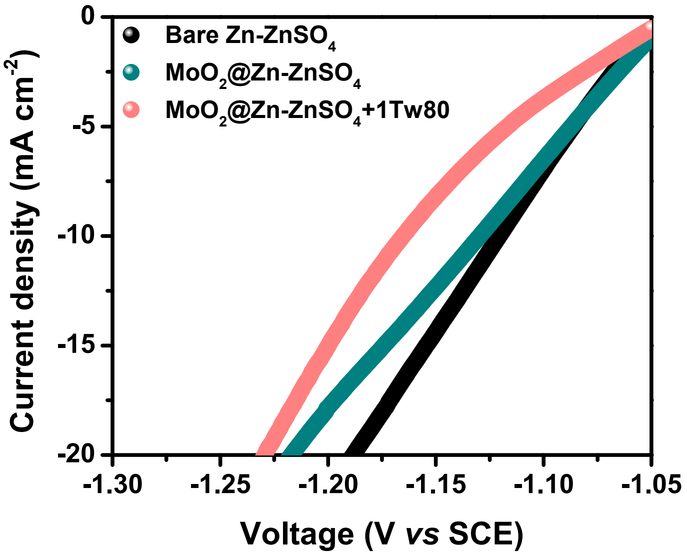

Figure S25. LSV curves of bare Zn and MoO_2@Zn in different electrolytes.

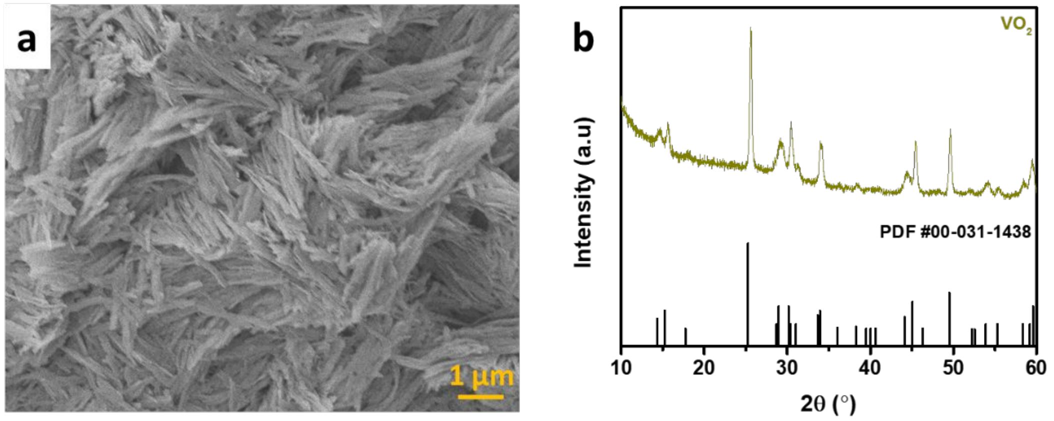

Figure S26. (a) SEM image and (b) XRD pattern of VO_2.

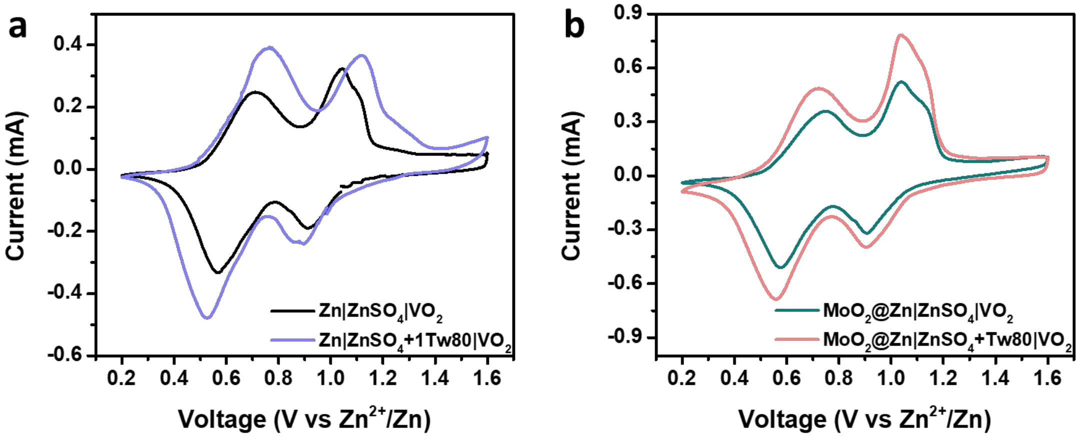

Figure S27. CV curves of Zn‖VO_2 full cells in different electrolytes applying (a) bare Zn anode and (b) MoO_2@Zn anode.

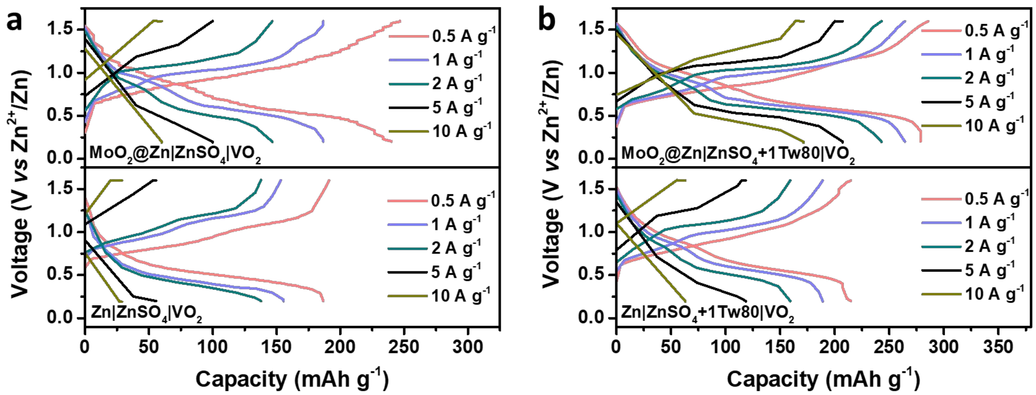

Figure S28. GCD curves under different current densities of Zn‖VO_2 and MoO_2@Zn‖VO_2 full cells using (a) $ZnSO_4$ and (b) $ZnSO_4$+1Tw80 electrolytes at a current density of 1 A g^{-1}.

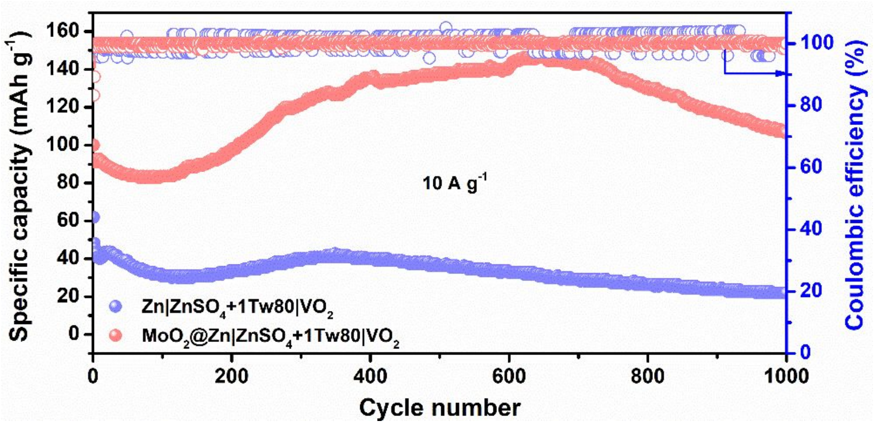

Figure S29. Cycling performance of Zn||VO_2 and MoO_2@Zn||VO_2 full cells using $ZnSO_4$+1Tw80 electrolyte at a high current density of 10 A g^{-1}.

Table S1. The comparison of the test conditions and electrochemical performance of our work with those previously reported by others.

Symmetric cell	Electrolyte	Current density (mA cm^{-2})	Capacity (mAh cm^{-2})	Lifespan (h)	Method	Ref.
NGO@Zn‖NGO@Zn	2 M $ZnSO_4$	1	1	1200	Surface modification	6
		5	5	300		
PPZ@Zn‖PPZ@Zn	2 M $ZnSO_4$	1	0.5	3000	Surface modification	7
		2	1	600		
		5	1	450		
PFPE@Zn‖PFPE@Zn	1 M $ZnSO_4$	0.25	0.25	800	Surface modification	8
		1	1	300		
ZrO_2@Zn‖ZrO_2@Zn	2 M $ZnSO_4$	1	0.5	1750	Surface modification	9
		10	1	860		
Zn-Cu alloy‖Zn-Cu alloy	2 M $ZnSO_4$	1	0.5	1050	Zn alloy anode	10
		3	2	410		
GaIn-Zn alloy‖GaIn-Zn alloy	2 M $ZnSO_4$	0.25	0.05	2125	Zn alloy anode	11
		1	0.1	1290		
		5	0.1	380		
3D Zn-CNT‖3D Zn-CNT	2 M $ZnSO_4$	2	2	200	3D-hosted Zn anode	12
		5	2.5	150		
3D Zn-Cu‖3D Zn-Cu	2 M $ZnSO_4$	0.5	0.5	350	3D-hosted Zn anode	13
3D Zn-CNF‖3D Zn-CNF	2 M $ZnSO_4$	0.25	0.25	350	3D-hosted Zn anode	14
		0.5	0.5	300		
		1	1	200		
Zn‖Zn	1 M $ZnSO_4$ + 1000 ppm CDA	0.5	0.5	1600	Electrolyte optimization	15
		1.5	0.5	800		
Zn‖Zn	3 M $ZnSO_4$ + 2 M LiCl	0.2	0.067	170	Electrolyte optimization	16
		1.5	0.5	170		

Zn‖Zn	1 M $ZnSO_4$ + 10 mM glucose	1	1	2000	Electrolyte optimization	17
		5	5	275		
Zn‖Zn	2 M $ZnSO_4$ + 300 ppm PVP	0.5	0.1	1000	Electrolyte optimization	18
		0.5	1	400		
		1	1	800		
UIO-66@Zn‖ UIO-66@Zn	3 M $ZnSO_4$ + 0.1 M $MnSO_4$	1	0.5	500	Surface modification and electrolyte optimization	19
		3	0.5	500		
		3	3	100		
PBC@Zn‖ PBC@Zn	1 M $ZnSO_4$ + 0.1 wt% $CuSO_4$	1	0.5	420	Surface modification and electrolyte optimization	20
MoO_2@Zn‖ MoO_2@Zn	1 M $ZnSO_4$ + 1 mM Tw80	1	1	6000	Surface modification and electrolyte optimization	This work
		5	5	705		
		10	5	125		

NGO = nitrogen-doped graphene; PPZ = zinc phosphate; PFPE = perfluoropolyether; CNT = carbon nanotube; CNF = carbon nanofiber; UIO-66 = Universistetet I Oslo-66 MOF; PBC = persimmon branch carbon; CDA = coconut diethanolamide; PVP = polyvinylpyrrolidone.

References

(1) Xue, X. Y.; Chen, Z. H.; Xing, L. L.; Yuan, S.; Chen, Y. J. SnO_2/α-MoO_3 Core-Shell Nanobelts and Their Extraordinarily High Reversible Capacity as Lithium-Ion Battery Anodes. *Chem. Commun.* **2011**, *47*, 5205–5207. https://doi.org/10.1039/c1cc00076d.

(2) Lou, X. W.; Zeng, H. C. Hydrothermal Synthesis of α-MoO_3 Nanorods via Acidification of Ammonium Heptamolybdate Tetrahydrate. *Chem. Mater.* **2002**, *14*, 4781–4789. https://doi.org/10.1021/cm0206237.

(3) Welter, E.; Chernikov, R.; Herrmann, M.; Nemausat, R. A Beamline for Bulk Sample X-Ray Absorption Spectroscopy at the High Brilliance Storage Ring PETRA III. *AIP Conf. Proc.* **2019**, *2054*, 040002. https://doi.org/10.1063/1.5084603.

(4) Chen, X.; Li, W.; Xu, Y.; Zeng, Z.; Tian, H.; Velayutham, M.; Shi, W.; Li, W.; Wang, C.; Reed, D.; Khramtsov, V. V.; Li, X.; Liu, X. Charging Activation and Desulfurization of MnS Unlock the Active Sites and Electrochemical Reactivity for Zn-Ion Batteries. *Nano Energy* **2020**, *75*, 104869. https://doi.org/10.1016/j.nanoen.2020.104869.

(5) Zhu, Y.; Matsumura, Y.; Velayutham, M.; Foley, L. M.; Hitchens, T. K.; Wagner, W. R. Reactive Oxygen Species Scavenging with a Biodegradable, Thermally Responsive Hydrogel Compatible with Soft Tissue Injection. *Biomaterials* **2018**, *177*, 98–112. https://doi.org/10.1016/j.biomaterials.2018.05.044.

(6) Zhou, J.; Xie, M.; Wu, F.; Mei, Y.; Hao, Y.; Huang, R.; Wei, G.; Liu, A.; Li, L.; Chen, R. Ultrathin Surface Coating of Nitrogen-Doped Graphene Enables Stable Zinc Anodes for Aqueous Zinc-Ion Batteries. *Adv. Mater.* **2021**, *33*, 2101649. https://doi.org/10.1002/adma.202101649.

(7) Wang, X.; Meng, J.; Lin, X.; Yang, Y.; Zhou, S.; Wang, Y.; Pan, A. Stable Zinc Metal Anodes with Textured Crystal Faces and Functional Zinc Compound Coatings. *Adv. Funct. Mater.* **2021**, *31*, 2106114. https://doi.org/10.1002/adfm.202106114.

(8) Tao, S.; Zhang, C.; Zhang, J.; Jiao, Y.; Li, M.; Lin, W.; Ran, L.; Clement, B.; Lyu, M.; Gentle, I.; Wang, L.; Knibbe, R. A Hydrophobic and Fluorophilic Coating Layer for Stable and Reversible Aqueous Zinc Metal Anodes. *Chem. Eng. J.* **2022**, *446*, 136607. https://doi.org/10.1016/j.cej.2022.136607.

(9) Liu, Y.; Guo, T.; Liu, Q.; Xiong, F.; Huang, M.; An, Y.; Wang, J.; An, Q.; Liu, C.; Mai, L. Ultrathin ZrO_2 Coating Layer Regulates Zn Deposition and Raises Long-Life Performance of Aqueous Zn Batteries. *Mater. Today Energy* **2022**, *28*, 101056. https://doi.org/10.1016/j.mtener.2022.101056.

(10) Kwon, M.; Lee, J.; Ko, S.; Lim, G.; Yu, S.-H.; Hong, J.; Lee, M. Stimulating Cu–Zn Alloying for Compact Zn Metal Growth towards High Energy Aqueous Batteries and Hybrid Supercapacitors. *Energy Environ. Sci.* **2022**, *15*, 2889–2899. https://doi.org/10.1039/D2EE00617K.

(11) Liu, C.; Luo, Z.; Deng, W.; Wei, W.; Chen, L.; Pan, A.; Ma, J.; Wang, C.; Zhu, L.; Xie, L.; Cao, X.-Y.; Hu, J.; Zou, G.; Hou, H.; Ji, X. Liquid Alloy Interlayer for Aqueous Zinc-Ion Battery. *ACS Energy Lett.* **2021**, *6*, 675–683. https://doi.org/10.1021/acsenergylett.0c02569.

(12) Zeng, Y.; Zhang, X.; Qin, R.; Liu, X.; Fang, P.; Zheng, D.; Tong, Y.; Lu, X. Dendrite-Free Zinc Deposition Induced by Multifunctional CNT Frameworks for Stable Flexible Zn-Ion Batteries. *Adv. Mater.* **2019**, *31*, 1903675. https://doi.org/10.1002/adma.201903675.
(13) Kang, Z.; Wu, C.; Dong, L.; Liu, W.; Mou, J.; Zhang, J.; Chang, Z.; Jiang, B.; Wang, G.; Kang, F.; Xu, C. 3D Porous Copper Skeleton Supported Zinc Anode toward High Capacity and Long Cycle Life Zinc Ion Batteries. *ACS Sustain. Chem. Eng.* **2019**, *7*, 3364–3371. https://doi.org/10.1021/acssuschemeng.8b05568.
(14) Jiang, Z.; Zhai, S.; Shui, L.; Shi, Y.; Chen, X.; Wang, G.; Chen, F. Dendrite-Free Zn Anode Supported with 3D Carbon Nanofiber Skeleton towards Stable Zinc Ion Batteries. *J. Colloid Interface Sci.* **2022**, *623*, 1181–1189. https://doi.org/10.1016/j.jcis.2022.05.058.
(15) Zhou, M.; Chen, H.; Chen, Z.; Hu, Z.; Wang, N.; Jin, Y.; Yu, X.; Meng, H. Nonionic Surfactant Coconut Diethanol Amide Inhibits the Growth of Zinc Dendrites for More Stable Zinc-Ion Batteries. *ACS Appl. Energy Mater.* **2022**, *5*, 7590–7599. https://doi.org/10.1021/acsaem.2c01048.
(16) Guo, X.; Zhang, Z.; Li, J.; Luo, N.; Chai, G.-L.; Miller, T. S.; Lai, F.; Shearing, P.; Brett, D. J. L.; Han, D.; Weng, Z.; He, G.; Parkin, I. P. Alleviation of Dendrite Formation on Zinc Anodes via Electrolyte Additives. *ACS Energy Lett.* **2021**, *6*, 395–403. https://doi.org/10.1021/acsenergylett.0c02371.
(17) Sun, P.; Ma, L.; Zhou, W.; Qiu, M.; Wang, Z.; Chao, D.; Mai, W. Simultaneous Regulation on Solvation Shell and Electrode Interface for Dendrite-Free Zn Ion Batteries Achieved by a Low-Cost Glucose Additive. *Angew. Chem. Int. Ed.* **2021**, *60*, 18247–18255. https://doi.org/10.1002/anie.202105756.
(18) Lin, C.; Liu, Y.; Zhang, X.; Miao, X.; Chen, Y.; Chen, S.; Zhang, Y. Regulating the Plating Process of Zinc with Highly Efficient Additive for Long-Life Zinc Anode. *J. Power Sources* **2022**, *549*, 232078. https://doi.org/10.1016/j.jpowsour.2022.232078.
(19) Liu, M.; Yang, L.; Liu, H.; Amine, A.; Zhao, Q.; Song, Y.; Yang, J.; Wang, K.; Pan, F. Artificial Solid-Electrolyte Interface Facilitating Dendrite-Free Zinc Metal Anodes via Nanowetting Effect. *ACS Appl. Mater. Interfaces* **2019**, *11*, 32046–32051. https://doi.org/10.1021/acsami.9b11243.
(20) Peng, J.; Yu, J.; Chu, D.; Hou, X.; Jia, X.; Meng, B.; Yang, K.; Zhao, J.; Yang, N.; Wu, J.; Li, L. Synergistic Effects of an Artificial Carbon Coating Layer and Cu^{2+}-Electrolyte Additive for High-Performance Zinc-Based Hybrid Supercapacitors. *Carbon* **2022**, *198*, 34–45. https://doi.org/10.1016/j.carbon.2022.07.012.
